# Incremental learning of humanoid robot behavior from natural interaction and large language models

**DOI:** 10.3389/frobt.2024.1455375

**Published:** 2024-10-10

**Authors:** Leonard Bärmann, Rainer Kartmann, Fabian Peller-Konrad, Jan Niehues, Alex Waibel, Tamim Asfour

**Affiliations:** Institute for Anthropomatics and Robotics (IAR), Karlsruhe Institute of Technology (KIT), Karlsruhe, Germany

**Keywords:** incremental learning, human–robot interaction, cognitive modeling, knowledge representation for robots, humanoid robots, large language models

## Abstract

Natural-language dialog is key for an intuitive human–robot interaction. It can be used not only to express humans’ intents but also to communicate instructions for improvement if a robot does not understand a command correctly. It is of great importance to let robots learn from such interaction experiences in an incremental way to allow them to improve their behaviors or avoid mistakes in the future. In this paper, we propose a system to achieve such incremental learning of complex high-level behavior from natural interaction and demonstrate its implementation on a humanoid robot. Our system deploys large language models (LLMs) for high-level orchestration of the robot’s behavior based on the idea of enabling the LLM to generate Python statements in an interactive console to invoke both robot perception and action. Human instructions, environment observations, and execution results are fed back to the LLM, thus informing the generation of the next statement. Since an LLM can misunderstand (potentially ambiguous) user instructions, we introduce incremental learning from the interaction, which enables the system to learn from its mistakes. For that purpose, the LLM can call another LLM responsible for code-level improvements in the current interaction based on human feedback. Subsequently, we store the improved interaction in the robot’s memory so that it can later be retrieved on semantically similar requests. We integrate the system in the robot cognitive architecture of the humanoid robot ARMAR-6 and evaluate our methods both quantitatively (in simulation) and qualitatively (in simulation and real-world) by demonstrating generalized incrementally learned knowledge.

## 1 Introduction

**FIGURE 1:**
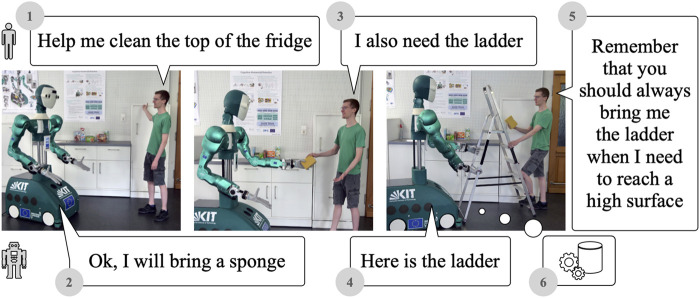
ARMAR-6 incrementally learns behavior from natural interactions. Demonstration videos can be found at https://lbaermann.github.io/interactive-incremental-robot-behavior-learning/.

Humans can easily communicate tasks and goals to a robot via language. Such a natural-language interface is key for achieving a truly intuitive human–robot interaction (HRI). However, the robot’s interpretation of such commands, and thus the resulting execution, might be sub-optimal, incomplete, or wrong. In such cases, it is desirable for the human to give further instructions to correct or improve the robot’s behavior. Furthermore, the robot should memorize the improvement strategy given by the human to incrementally learn from them and thus avoid the same mistake in the future. For instance, consider the interaction depicted in Figure 1. First, the user instructs the robot to help him clean the top of the fridge (1). The robot then executes several actions to hand over a sponge to the human (2). The user observes this insufficient result and gives instructions for improvement (“I also need a ladder”) (3), whereupon the robot performs corrective actions (4). If the desired goal is achieved, the user can reconfirm the correction (5), which leads to the robot updating its memory appropriately (6), thus incrementally learning new behavior based on language instructions.

In this paper, we present a system to achieve such behavior and describe its implementation on the humanoid robot ARMAR-6 (Asfour et al., 2018). We build on the capabilities of large language models (LLMs) (Brown et al., 2020; Touvron et al., 2023; OpenAI, 2023a; OpenAI, 2023b) emerging from massive-scale next token prediction pre-training, and aim to transfer their success to HRI. The goal is to utilize the rich world knowledge contained in LLMs for an embodied natural language dialog, thus enhancing the capabilities of the LLM by integrating robot perception and action. In the cognitive architecture of our humanoid robot (Peller-Konrad et al., 2023), this means the LLM will be in charge of high-level planning and decision-making. Recent works like SayCan (Ahn et al., 2022) and Code as Policies (CaP) (Liang et al., 2023) already demonstrate the usefulness of applying LLMs to orchestrate robot abilities, enabling high-level task understanding, planning, and generalization. Going a step further, inner monologue (Huang et al., 2022b) feeds back execution results and observations into the LLM, thus involving the LLM in a closed-loop interaction.

Inspired by these works, we propose to utilize the code-writing capabilities of LLMs to directly integrate it into closed-loop orchestration of a humanoid robot. This is achieved by simulating an interactive (Python) console in the prompt and letting the LLM produce the next statement, given the previous execution history, including results returned or exceptions thrown by previous function calls. Thus, the LLM can dynamically respond to unexpected situations such as execution errors or wrong assumptions while still leveraging the power of a code-based interaction such as storing results in intermediate variables or defining new functions.

For utilizing the few- and zero-shot capabilities of LLMs, it is crucial to design (a set of) prompts to properly bias the LLM toward the desired output. All of the above works use a predefined, manually written set of prompts tuned for their respective use case. However, no LLM or prompting scheme will always interpret each user instruction correctly, especially since natural language can be ambiguous and correct execution might depend on user preferences. Therefore, we propose a novel, self-extending prompting method to allow incremental learning of new behaviors and adaptation of existing high-level behaviors. To this end, our system dynamically constructs prompts based on a set of interaction examples populated from the robot’s prior knowledge and previously learned behavior. Given a user instruction, we rank all such interaction examples by semantic similarity to the input and select the top- k entries to construct the actual prompt to the LLM. Crucially, the robot’s prior knowledge contains specific examples involving the user complaining about mistakes and correcting the robot or instructing it on how to improve its behavior. Therefore, when the system fails to correctly execute a task and the user gives such corrective instructions, the LLM is biased to invoke code that inspects the current execution history and forwards it to another, few-shot-prompted LLM. This LLM can inspect the complete interaction, including all user inputs, performed actions, and observed results, represented as the transcript of an interactive Python console. It then spots the mistakes and produces an improved interaction using chain-of-thought (CoT) prompting (Wei et al., 2022). Finally, the improved transcript will be added to the interaction examples, thus enabling the system to perform better the next time a similar task is requested.

Our method is explained in detail in Section 3. We evaluate our system quantitatively on the scenarios defined in CaP (Liang et al., 2023) to show the effectiveness of our proposed approach in Section 4. Furthermore, Section 5 demonstrates the capabilities of incremental learning from natural-language interactions on a real-world humanoid robot. Our code can be found at https://github.com/lbaermann/interactive-incremental-robot-behavior-learning.

## 2 Related work

We start by reviewing works on understanding and learning from natural language in robotics. Subsequently, we present works using LLMs for high-level orchestration of robot abilities. Finally, we focus on dynamic creation of prompts for LLMs.

### 2.1 Understanding and learning from natural language

Understanding and performing tasks specified in natural language has been a long-standing challenge in robotics (Tellex et al., 2020). *Grounding* the words of natural-language sentences in the robot’s perception and action is a major challenge known as the *signal-to-symbol gap* (Krüger et al., 2011). Many works have focused on the grounding of expressions referring to objects, places, and robot actions based on graphical models (Tellex et al., 2011; Misra et al., 2016), language generation (Forbes et al., 2015), or spatial relations (Guadarrama et al., 2013), especially for ambiguity resolution (Fasola and Matarić, 2013; Shridhar et al., 2020). Pramanick et al. (2020) focused on resolving task dependencies to generate execution plans from complex instructions. However, in these works, the robot does not explicitly learn from language-based interactions. In contrast, Walter et al. (2013) enriched the robot’s semantic environment map from language, and Bao et al. (2016) syntactically parsed daily human instructions to learn attributes of new objects. In Kartmann and Asfour (2023), the robot asked for a demonstration if its current understanding of a spatial relation is insufficient to perform a given instruction. Other works go further by learning on the task level. Mohan and Laird (2014) learned symbolic task representations from a language interaction using explanation-based learning. Nicolescu et al. (2019) learned executable task representations encoding sequential, non-ordering, or alternative paths of execution from verbal instructions for interactive teaching by demonstration. Weigelt et al. (2020) considered the general problem of programming new functions on code level via natural language. Although our goal is similar to that of these works, we leverage LLMs for task-level reasoning and learning.

### 2.2 Code generation and interaction with LLMs

Generating code from natural-language specifications is a large area of active research. For instance, LLMs tuned specifically on code (Chen et al., 2021; Nijkamp et al., 2023) perform well in common code-generation benchmarks. Madaan et al. (2022b) showed that code-based models have more structured representations, thus aiding structured (e.g., graph-based) tasks. Training code-LLMs can also benefit from using an interpreter in the optimization loop (Le et al., 2022; Haluptzok et al., 2023). We refer the reader to recent surveys (Zheng et al., 2024; Ahmed et al., 2023; Dehaerne et al., 2022; Wang and Chen, 2023) for a more in-depth discussion.

Another recent trend is to use LLMs in an interactive, chat-style format. This became popular through OpenAI’s models (OpenAI, 2023a,b) and is typically powered by fine-tuning on alignment data using reinforcement learning from human feedback (Ouyang et al., 2022). In a code-based setting, such an interaction can, for instance, assist software development (Lahiri et al., 2023; Google, 2023). Furthermore, many recent works utilize interactive coding strategies to deploy LLMs as agents (Yang et al., 2024). For instance, Voyager (Wang G. et al., 2024) iteratively learns to master the game of Minecraft by letting an LLM code functions, and InterCode (Yang et al., 2023) connects an LLM to a Bash shell to solve a file system task, similar to our use of an interactive Python console. Recent benchmarks (Liu et al., 2024; Wang X. et al., 2024) will further catalyze this development. We deploy such an interactive coding strategy to real-world humanoid robotics and enrich it with incremental learning from natural interactions.

### 2.3 Orchestrating robot behavior with LLMs

Recently, many works extended the capabilities of LLMs by giving them access to external models, tools, and APIs (Mialon et al., 2023; Parisi et al., 2022; Qin et al., 2023; Wang et al., 2023). Tool usage can also be combined with reasoning techniques such as CoT prompting (Wei et al., 2022) to significantly improve planning (Yao et al., 2023). In particular, orchestrating robot behavior and thus interacting with the physical environment can be seen as an embodied special case of LLM tool usage. Huang et al. (2022a) initially proposed the idea to utilize world knowledge from LLM pre-training to map high-level tasks to executable mid-level action sequences. SayCan (Ahn et al., 2022) fuses LLM output probabilities with pre-trained affordance functions to choose a feasible plan, given a natural language command. Socratic models (Zeng et al., 2023) combine visual and textual LLMs to generate instructions in the form of API calls, which are then executed by a pre-trained language-conditioned robot policy. Both Code as Policies (CaP) (Liang et al., 2023) and ProgPrompt (Singh et al., 2023) demonstrate the usefulness of a code-generating LLM for robot orchestration as they convert user commands to (optionally, recursively defined) policy code grounded in predefined atomic API calls. Although the generated policies can react to the robot’s perception, these approaches do not directly involve the LLM in the online execution of a multi-step task after the policy has been generated. In contrast, Inner Monologue (Huang et al., 2022b) feeds back execution results and observations into the LLM, but it does not rely on code-writing, thus missing its combinatorial power. KnowNo (Ren et al., 2023) iteratively asks the LLM for a set of possible next steps, determines the LLM’s confidence in each possibility using its output token distribution in a multiple-choice setup, and then uses conformal prediction to decide whether the system is sure how to proceed or should ask the user for help. AutoGPT+P (Birr et al., 2024) combines an LLM with a symbolic planner. Recent technical reports (Vemprala et al., 2023; Wake et al., 2023) provide guidance on utilizing ChatGPT (OpenAI, 2023a) for robot orchestration. Meanwhile, TidyBot (Wu et al., 2023) uses GPT-3 (Brown et al., 2020) in a similar way to generate high-level plans for tidying up a cluttered real-world environment, but the authors focus on personalization by summarizing and thereby generalizing individual object placement rules.

With our proposed emulated Python console prompting, we differ from these existing works by 1) formatting and interpreting all interactions with the LLM as Python code, in contrast to Ahn et al. (2022) and Huang et al. (2022b); 2) closing the interaction loop by enabling the LLM to reason about each perception and action outcome, in contrast to Liang et al. (2023), Singh et al. (2023), Wake et al. (2023), Zeng et al. (2023), and Ahn et al. (2022); 3) allowing the LLM to decide when and which perception primitives to invoke, instead of providing a predefined list of observations (usually a list of objects in the scene) as part of the prompt as in Zeng et al. (2023), Huang et al. (2022b); Singh et al. (2023), Liang et al. (2023), and Wu et al. (2023); and 4) simplifying the task for the LLM by allowing it to generate one statement at a time, in contrast to Liang et al. (2023), Singh et al. (2023), and Vemprala et al. (2023).

### 2.4 Dynamic prompt creation

When prompting an LLM to perform a task, quality and relevance of the provided few-shot examples are key to the performance of the system. Thus, several works propose to dynamically select these examples (e.g., from a larger training set) for constructing a useful prompt. Liu et al. (2022) improved the performance in a downstream question-answering (QA) task by selecting relevant few-shot samples via k -nearest-neighbor search in a latent space of pre-trained sentence embeddings (Reimers and Gurevych, 2019) representing the questions. Ye et al. (2023) selected not only the most similar but also a diverse set of samples. Luo et al. (2023) showed that this dynamic prompt construction is also applicable for instruction-fine-tuned language models (LMs) (Ouyang et al., 2022) and in combination with CoT prompting. Song et al. (2023) used top- k retrieval for instructing an LLM to plan robotic tasks. Similar to that approach, we apply vector embeddings of human utterances to find the top- k examples that are most similar to the current situation.

Other works go further by proposing to update the database of examples by user interactions. In Madaan et al. (2022a), GPT-3 was tasked with solving lexical and semantic natural language processing questions few-shot by generating both an understanding of the question and the answer. A user can then correct an erroneous understanding to improve the answer, and such a correction is stored in a lookup table for later retrieval on similar queries. Similarly, user feedback can be used to improve open-ended QA by generating an entailment chain along with the answer and allowing the user to then correct false model beliefs in that entailment chain (Dalvi Mishra et al., 2022). Corrections are stored in memory and later retrieved based on their distance to a novel question.

In our work, we also propose to store corrective user feedback as interaction examples in the robot’s memory. However, we go even further by 1) letting the LLM decide when such feedback is relevant (by invoking a certain function), 2) generating new examples of improved behavior from the human’s feedback, and thus, 3) treating prior knowledge and instructed behavior in a uniform way by treating both as interaction examples in the robot’s memory. Vemprala et al. (2023) mentioned that ChatGPT can be used to change the code based on high-level user feedback. However, they do not combine this with incremental learning to persist the improved behavior.

**FIGURE 2:**
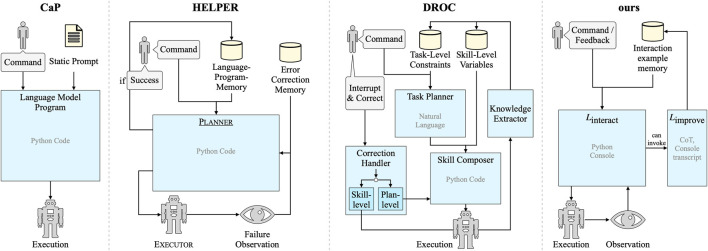
Comparison of Code as Policies (Liang et al., 2023), HELPER (Sarch et al., 2023), DROC (Zha et al., 2023), and our method, focusing on information flow from user input, observations, prompts, and memories to LLM modules to robot execution, and how the methods learn from user interactions. Building on the interactive Python console prompting scheme, our method realizes incremental learning from natural interactions in a conceptually simple way.

Closest to our approach are the concurrent works DROC (Zha et al., 2023) and HELPER (Sarch et al., 2023), shown in Figure 2. Similar to our learning from the interaction, DROC (Zha et al., 2023) distills knowledge from problematic interactions and retrieves it later when solving new tasks. Although the goal and problem setting are similar, we differ by formulating the complete interaction as code instead of separating task-level and skill-level into natural-language- and code-level interaction, respectively, and also by generalizing incremental learning as code manipulation instead of explicitly memorizing task-level natural-language constraints and skill-level variable assignments separately. HELPER (Sarch et al., 2023) retrieves few-shot examples for the LLM’s prompt from a language-program memory similar to our interaction example memory and learns personalized robot behavior by extending the memory. In contrast to our approach, they add examples only from successful episodes, and they have separate mechanisms for normal behavior and error correction. We focus on learning from feedback in erroneous or suboptimal episodes, and we treat initial and follow-up instructions uniformly using the proposed Python console prompting.

## 3 Approach

In this section, we more precisely formulate the considered problem and explain our approach to intuitive HRI and incremental learning of humanoid robot behavior using LLMs.

### 3.1 Problem formulation and concept

**FIGURE 3:**
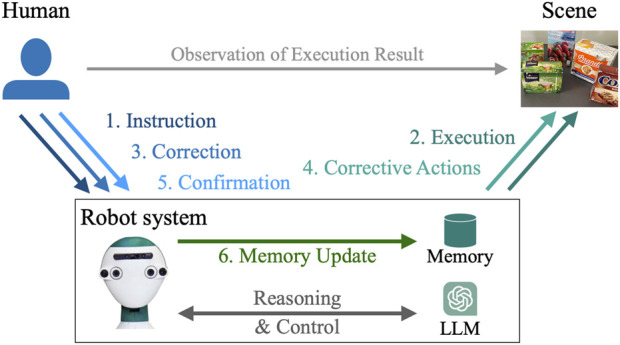
Incremental learning of robot behavior from interactions.

In this work, we consider the problem of enabling a robot to interact with a human in natural language, as depicted in Figure 3. First, the human gives a natural-language instruction to the robot. Then, the robot interprets the instruction and performs a sequence of actions. However, the performed actions might be sub-optimal, incomplete, or wrong. In that case, the human instructs the robot how to improve or correct its behavior. The robot executes further actions accordingly, and if the human is satisfied with the result, they can confirm that the robot should memorize this behavior. Finally, the robot must incrementally learn from the corrective instructions and avoid similar mistakes in the future.

We formulate this problem as follows: Consider a robot with a set of functions $F=\left\{F_{1},\ldots ,F_{n}\right\}$ . A function can be invoked to query the robot’s perception or execute certain actions. Furthermore, let M denote the knowledge of interactions and behaviors as part of the episodic memory of the robot, which is initialized by prior knowledge. Based on the initial instruction $I_{0}$ and M , the robot must perform a sequence of function invocations $\left(f_{1},\ldots ,f_{m}\right)$ , where each invocation $f_{i}$ consists of the invoked function $F_{i}$ with its corresponding parameters. Executing these invocations yields a sequence of results $\left(r_{1},\ldots ,r_{m}\right)$ . Overall, performing the task indicated by $I_{0}$ results in an *interaction history* H of the form

$$H=\left(\left(f_{1},r_{1}\right),\ldots ,\left(f_{m},r_{m}\right)\right)\leftarrow perform\left(I_{0},M\right).$$ (1)

Note that we explicitly allow executing a generated invocation right away (potentially modifying the world state W ) and using the result to inform the generation of the subsequent invocation. Therefore, the current history $H_{t}=\left(\left(f_{1},r_{1}\right),\ldots ,\left(f_{t},r_{t}\right)\right)$ is available when generating the next invocation $f_{t+1}$ , i.e., for $t\in \left\{0,\ldots ,m-1\right\}$ ,

$$f_{t+1}\leftarrow generate\left(I_{0},H_{t},M\right),$$ (2)

$$\left(r_{t+1},W_{t+1}\right)\leftarrow execute\left(f_{t+1},W_{t}\right),$$ (3)

$$H_{t+1}\leftarrow H_{t}◦\left(\left(f_{t+1},r_{t+1}\right)\right),$$ (4)

where ◦ denotes sequence concatenation. In other words, invocations are generated auto-regressively by reasoning over the memory, the instruction, and the previous actions and their execution results.

To unify the subsequent notation, we define the human’s instructions as a special case of perception, i.e., the system perceives them as a result of invoking the function $F_{wait}\in F$ . Using that terminology, $H_{0}=\left(\left(f_{\text{wait}},I_{0}\right)\right)$ , and we can drop $I_{0}$ as an explicit parameter of generate . Similarly, further instructions are handled as part of the interaction history.

If the human gives an instruction to correct the robot’s behavior, the robot must be able to learn from this instruction to improve its behavior in the future. We model this capability as another function $F_{learn}\in F$ . Its purpose is to update the robot’s interaction knowledge M to learn from the corrective instructions and avoid the mistake in the future:

$$M\leftarrow learn\text{\_}from\text{\_}interaction\left(M,H_{t}\right),$$ (5)

where $H_{t}$ is the interaction history when $F_{learn}$ is called.

**FIGURE 4:**
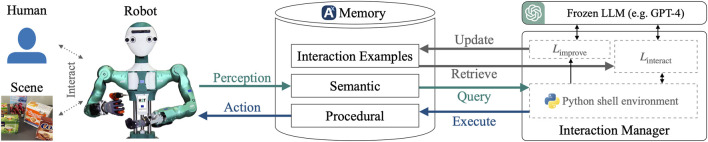
Conceptual view of our system. The robot’s memory system (Peller-Konrad et al., 2023) works as a mediator between the interaction manager and the robot system. The interaction LLM acts in a Python console environment. It can invoke functions to fetch the content of the current scene (as given by perception modules and stored in the memory) or invoke skills and thus perform robot actions. Relevant interaction examples are queried from the memory for few-shot prompting of the LLM. Incremental learning is performed by an improvement LLM updating the interaction example memory with new content learnt from instructions.

To address this problem, we propose a system as depicted in Figure 4. A humanoid robot is interacting with a human and the scene. The robot is equipped with a multimodal memory system containing the following information about the current scene: first, semantic knowledge about objects, locations, and agents in the world; second, sub-symbolic and symbolic knowledge about the current scene, including object relations; third, the procedural memory of the robot, containing executable skills (in our case, implemented through scripted policies). An execution request sent to the procedural memory triggers physical robot actions. The set of available functions F contains functions to query the semantic and procedural memory. Finally, we implement M as part of the episodic memory of the robot containing interaction histories H , i.e., short episodes of interactions between the human and the robot, including the natural-language inputs, the actions executed by the robot, and their results.

The *interaction manager* is responsible for the high-level orchestration of the robot’s abilities. It has access to two instances of LLMs, an *interaction LLM* $L_{interact}$ and an *improvement LLM* $L_{improve}$ , as well as a Python console environment E to execute generated function invocations. Utilizing E , we uniformly represent all H∈M and $H_{t}$ as a textual Python console transcript, i.e., a sequence of function invocations $f_{i}$ represented as the Python statement and return values $r_{i}$ converted to text using Python’s “repr” function. $L_{interact}$ is prompted by the interaction manager with the available functions F , the current interaction history $H_{t}$ , and relevant few-shot examples retrieved from M , and it generates function invocations f . Following the notation of Equations 2, 3, the function generate is implemented through $L_{interact}$ , while the function execute is provided by E . By generating an invocation of $F_{learn}\in F$ , $L_{interact}$ can trigger Equation 5. We implement the function learn_from_interaction by few-shot prompting $L_{improve}$ . It reasons over $H_{t}$ and generates an improved version of the interaction, which is then saved to the memory M .

### 3.2 Procedure overview

**FIGURE 5:**
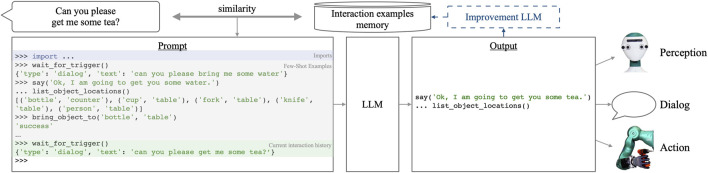
Overview of our method for incremental learning of robot behavior. We use an LLM [in our experiments, GPT-4 (OpenAI, 2023b)] to control robot perception and action, given a prompt of few-shot examples (bottom, Section 3.3). Prompts are constructed dynamically based on the similarity to the current user request (top left, Section 3.4). The interaction example memory is initialized with prior knowledge and then incrementally enriched by LLM-improved problematic interactions to learn from mistakes (top right, Section 3.5).

To start, we populate the memory M with both prior knowledge (i.e., predefined interaction examples) and previously learned interaction examples. The interaction manager sets up E , including F , and then invokes an initial $F_{wait}=$ “wait_for_trigger()” inside that environment. This call waits for dialog input and returns when the human gives an initial instruction. The interaction manager handles any function return value by inserting its textual representation into the current interaction history, thus extending $H_{t}$ . Thereby, it emulates the look of a Python console (Section 3.3). In the following, a prompt is constructed (Section 3.4) based on F , the most relevant examples from M , and $H_{t}$ . This prompt is passed to $L_{interact}$ to produce the next command(s). The generated code is executed within E , and both the code and its return values are again inserted into $H_{t}$ . The interaction manager repeats this process as the high-level behavior-driving loop of the robot (see Figure 5). Note that $L_{interact}$ can listen to further user utterances by generating “wait_for_trigger()” again. Our proposed prompt-based incremental learning strategy (Section 3.5) is also invoked by $L_{interact}$ itself when it calls $F_{learn}=$ “learn_from_interaction()”.

### 3.3 LLM interacting with an emulated Python console

The left of Figure 5 shows an interaction example using our proposed prompting scheme emulating a Python console. All commands entered into the emulated console (lines starting with “>>>” or “…”) are to be generated by the LLM, while the function return values are inserted below each invocation. The proposed syntax enables a closed interaction loop so that the LLM can dynamically react to unexpected situations and errors while also keeping the flexibility of coding non-trivial statements. We achieve this by setting “>>>” to be the stop token when prompting the LLM. This means that the LLM can generate continuation statements (including control flow and function definitions) by starting a new line with “…”. Since generation stops at the beginning of the next statement, the LLM’s output will also include the expected outcome of its own command, which we discard for the scope of this work.

During our experiments, we observed that it is important for functions to provide semantically rich error messages, including hints on how to improve. This leads to self-correcting behavior (Skreta et al., 2023). For instance, when calling “move_to” with an invalid or underspecified location such as “counter,” we pass the error message “invalid location. Use one of the locations returned by list_locations()” to the LLM. In this example, the error message guides the LLM to query a list of possible locations, which are then used to correctly ground the natural-language request to the name “inFrontOf_mobile-kitchen-counter_0” that the “move_to” function understands.

Analogously to Code as Policies (Liang et al., 2023), we dynamically generate non-existing functions that the LLM tries to use. Specifically, when $L_{interact}$ generates code that refers to an undefined function, we invoke another LLM, $L_{\mathrm{fgen}}$ , that is prompted to define the function, given the line of code that is using it as the context. For $L_{\mathrm{fgen}}$ , we exactly follow the method of Liang et al. (2023), including recursive function generation. The generated function is then inserted into the emulated Python console *before* the statement that referred to the undefined function, and then, that statement is executed. The purpose of inserting the function definition into the execution history is that it is thereby accessible to user feedback and can be improved upon by incremental learning.

### 3.4 Dynamic prompt construction

We dynamically construct the prompt for $L_{interact}$ depending on the current interaction history $H_{t}$ (i.e., the code statements, execution results, and user inputs observed so far). We start with some predefined base prompt, stating the general task and “importing” all defined names and functions. These imports are generated dynamically, given the symbols defined in E , i.e., the available functions F . The second part of the prompt consists of few-shot examples. For this, we make use of a memory M of coding interaction examples, where each entry follows the Python console syntax defined in Section 3.3. M is initialized with hand-written prompts, but it is later extended dynamically, as explained in Section 3.5. Given the current interaction history $H_{t}$ , we define a similarity measure $S\left(H,H_{t}\right)$ , see below, for each H∈M and choose the top- kH to become part of the actual prompt. Afterward, $H_{t}$ itself is inserted into the prompt to provide the LLM with the current context. Finally, the prompt is completed by inserting a syntax trigger for the LLM to correctly generate the next command, i.e., “>>>.” An example can be seen on the left of Figure 5.

To implement the similarity function $S\left(H,H_{t}\right)$ , we assume that examples with comparable natural-language instructions are helpful. Therefore, we extract all such instructions from $H_{t}$ and each H∈M . In our specific Python-console-based representation, this means that we search for function calls that trigger user interaction (“ask,” “wait_for_trigger”) and extract their respective return values. Let $I_{t}^{i}$ with i=1,…,N denote the N most recent instructions in $H_{t}$ (where $I_{t}^{1}$ is the most recent one), and $I_{H}^{j}$ with $j=1,\ldots ,M_{H}$ denote all the $M_{H}$ instructions found in each H∈M . We make use of a pre-trained sentence-embedding model (Reimers and Gurevych, 2019) to measure the semantic similarity $sim\left(a,b\right)=E\left(a\right)\cdot E\left(b\right)$ between two natural language sentences a,b by the dot product of their latent space embeddings $E\left(\cdot \right)$ . First, we compute a latent representation of $H_{t}$ as

$$e_{t}=\overset{N}{\underset{i=1}{\sum }}\gamma^{i-1}E\left(I_{t}^{i}\right),$$ (6)

where γ=0.6 is an empirically chosen decay factor. Then, we determine a score $\alpha_{H}^{j}$ for each instruction $I_{H}^{j}$ of each history H∈M , as given by

$$\alpha_{H}^{j}=e_{t}\cdot E\left(I_{H}^{j}\right).$$ (7)

The final similarity score is given by $S\left(H,H_{t}\right)=\max_{j}\alpha_{H}^{j}$ , and we pick the top- k such H as the few-shot examples for the prompt.

### 3.5 Incremental prompt learning

To enable our system to learn new or improved behavior from user interaction, we propose to make M itself dynamic. For this purpose, we introduce a special function $F_{learn}=$ “learn_from_interaction().” This function is always “imported” in the base prompt, and there are predefined code interaction examples $H_{\text{learn}}\in M$ involving this call. These $H_{\text{learn}}$ will be selected by dynamic prompt construction if semantically similar situations occur. They involve failure situations, where the user has to tell the robot what and how to improve and that it should do better next time. Thus, when a mistake occurs and the user complains, these examples will be selected for the prompt, and $L_{interact}$ is biased toward invoking $F_{learn}$ .

Listing 1 Example of the LLM-transcript generated by a “learn_from_interaction()” call. The parts starting with “**LLM**” are generated by theLLM, while the “**Prompt**” parts are fixed prompts (and the input code snippet to improve). A full prompt including few-shot examples isprovided in Supplementary Appendix G.

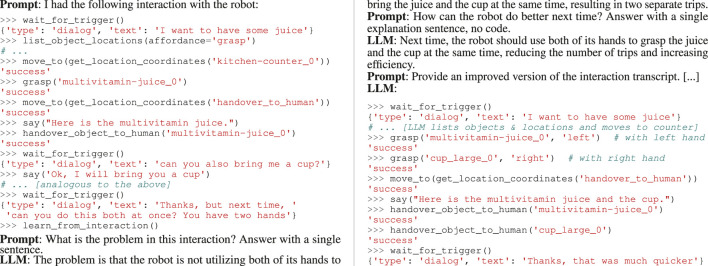

To implement learning from an erroneous interaction $H_{t}$ , we query $L_{improve}$ in a CoT manner to identify and fix the problem. Specifically, we provide $H_{t}$ and first ask for a natural-language description of the problem in this interaction. Subsequently, we request $L_{improve}$ to explain what should be improved next time. Finally, $L_{improve}$ is asked for an improved version $H_{t}^{*}$ of the interaction (in the given Python console syntax), and $H_{t}^{*}$ is added to the memory M . In that way, the next time a similar request occurs, $H_{t}^{*}$ will be selected by dynamic prompt construction, and $L_{interact}$ is biased toward not making the same mistake again. An example LLM transcript of such $F_{learn}$ implementation can be found in Listing 1. For robustness, there are three cases where we discard the generated $H_{t}^{*}$ : first, we ignore the call to $F_{learn}$ if it does not follow immediately after a user utterance since we only want to learn from explicit human feedback. Second, we abort the learning if the response to the first CoT request is that there is no problem. Third, if $H_{t}^{*}$ is equal to the input interaction $H_{t}$ , we discard it.

## 4 Simulated evaluation

### 4.1 Experimental setup

To quantitatively assess the performance of our method, we utilize the evaluation protocol from Code as Policies (Liang et al., 2023), involving a simulated tabletop environment with a UR5e arm and Robotiq 2F85 gripper manipulating a set of blocks and bowls of 10 different colors. We use their seven seen and six unseen instructions (SI/UI), where each instruction is a task with placeholders that are filled with attributes (e.g., “pick up the < *block* > and place it on the < *corner* > ”). The set of possible attribute values is also split into seen and unseen attributes (SA/UA). For more details, refer to Liang et al. (2023).

As our focus is on incremental learning from natural-language interactions, our methodology involves human supervision as follows: we first set up a randomly generated scene and pass the instruction to the evaluated system. The system generates some code that utilizes the same API as in Liang et al. (2023). Specifically, there are “perception” functions (utilizing the ground-truth simulation state) to query all object names and positions and convert normalized to absolute coordinates and an “action” function to move an object to another object or position. For details, see Supplementary Appendix A or Liang et al. (2023). During code execution, the human observes the robot’s actions by watching the simulation rendering. Each run can result in success (goal reached), failure (goal not reached), error (system threw unhandled exception), or timeout (e.g., system got stuck in a loop). The latter two lead to immediate termination of the experiment. In contrast, when the system yields control normally (after code execution for CaP and on $F_{wait}$ for our method), the resulting world state is checked using scripted ground-truth evaluation functions, leading to either success or failure outcomes. The human is then presented with this outcome and has the option to provide feedback or improvement instructions to the robot, which are again passed to the system. The success detection is performed every time the system yields control, and the sequence of states and user interactions is recorded. Note that we allow user feedback even when already in the success state as the execution might still have been suboptimal and the human may want to provide feedback to learn from for next time. Details and example interactions can be found in Supplementary Appendix B.

Every task is repeated 10 times using randomly generated scenes, and each run is performed in sequence, i.e., the interaction memory is not reset between runs in order to allow for incremental learning. To assess the results, we compute the following metrics from the execution traces:

*s* is the turnout success rate, i.e., the percentage of runs that ended in the success state (optionally after user interaction that clarifies the goal or helps the system).
*i* is the initial success rate, i.e., the percentage of runs that yielded a successful state on the first system return, i.e., where no user interaction was required to reach success.
*n* counts the number of user interactions that were required until the success state was first reached. For runs that count into the initial success category, n=0 , while for non-successful runs, n is undefined. When aggregating n , we average only the runs that ended successfully.

### 4.2 Baselines and methods

CaP: We utilize the prompts provided by Liang et al. (2023). This is equivalent to our system without incremental learning and without the interactive console formatting. Specifically, we note that CaP has no way of feeding back coding errors to the system, i.e., it fails immediately if the generated code is syntactically invalid or throws an exception.

HELPER: we adapt the code and prompts provided by Sarch et al. (2023) to the simulated tabletop evaluation scenario and API. For few-shot example retrieval, we set k=16 for a fair comparison. Specifically, we feed back execution errors to the *self-reflection and correction* prompt, and user feedback is passed as a new command to the Planner. HELPER’s few-shot memory is expanded with successful trials. Further details can be found in Supplementary Appendix A.

Dynamic CaP: To make CaP a more competitive baseline, we add a simple form of learning and top- k retrieval and call this method *Dynamic CaP*. Similar to HELPER and our method, Dynamic CaP uses a memory of few-shot samples and stores code transcripts of successful episodes as new samples therein. On every request, we fill the prompt with the top- k similar examples retrieved from the memory. Further implementation details can be found in Supplementary Appendix A.

Ours: this is our full system with incremental learning and a value of k=16 for few-shot sample retrieval. We split and translated the 16 samples from the CaP prompts into our interactive console syntax to initialize the memory of interaction examples. Furthermore, there are two very short samples that demonstrate when to call $F_{learn}$ .

Ours w/o learning: This is our system, but without incremental learning. k=16 means that all samples are used as the interaction example memory is static.

Ours w/o retrieval: This is our system with incremental learning but a very high value of k=64 for few-shot sample retrieval, which effectively is a system that does not use retrieval. Note that the prompt construction is still dynamic as the order of the samples is determined by the similarity to the current request (cf. Section 3.4).

Furthermore, we compare the differently capable LLMs gpt-3.5-turbo-0301 and gpt-4-0613 of the OpenAI API (OpenAI, 2023a,b). For $L_{\mathrm{improve}}$ , we always use GPT-4. We note that the original CaP numbers (Liang et al., 2023) were reported with the codex model (Chen et al., 2021) that is no longer available. We reproduced their experiments with the newer models but did not perform further prompt tuning; therefore, our success rates for CaP are lower than those reported in Liang et al. (2023). Specifically, GPT-3.5 sometimes generates natural-language responses instead of code, which causes CaP to fail with a SyntaxError.

### 4.3 Results

Tables 1, 2 present the aggregated results of our experiments, while further details can be found in Supplementary Appendix C. From these results, we draw the following main insights:

**TABLE 1:** Evaluation results on simulated tabletop tasks: success rate s and initial success rate i .

	Test	Ours			HELPER	Dyn. CaP	CaP
		Full	w/o retrieval	w/o learning			
		s i	s i	s i	s i	s i	s i
GPT-4	SA SI	100 97.5	97.5 90.0	98.8 90.0	97.5 87.5	88.8 86.2	85.0 71.2
	UA SI	100 92.5	98.8 95.0	98.8 92.5	100 93.8	97.5 93.8	96.2 81.2
	UA UI	93.3 85.0	91.7 81.7	91.7 78.3	91.7 81.7	63.3 46.7	53.3 35.0
GPT-3.5	SA SI	95.0 87.5	93.8 82.5	85.0 43.8	93.8 77.5	57.5 55.0	53.8 52.5
	UA SI	97.5 86.2	96.3 88.8	80.0 45.0	87.5 71.2	65.0 57.5	60.0 58.8
	UA UI	85.0 70.0	56.7 51.7	66.7 43.3	80.0 50.0	46.7 36.7	16.7 15.0

**TABLE 2:** Evaluation results on simulated tabletop tasks: average number of interactions until success n .

	Test	Ours			HELPER	Dyn. CaP	CaP
		Full	w/o retrieval	w/o learning			
GPT-4	SA SI	0.04	0.12	0.37	0.21	0.06	0.26
	UA SI	0.14	0.12	0.1	0.1	0.07	0.35
	UA UI	0.16	0.18	0.55	0.22	0.62	0.74
GPT-3.5	SA SI	0.14	0.25	1.09	0.31	0.16	0.02
	UA SI	0.33	0.15	0.95	0.38	0.23	0.06
	UA UI	0.28	0.19	1.29	0.68	0.48	0.07

**Interactive feedback helps achieve success**. For all methods, s is notably above i , which means that $L_{\mathrm{interact}}$ effectively uses human feedback to improve its behavior. This effect is less stressed for CaP with GPT-3.5 as it often immediately fails with an error, thus not allowing for further interaction.

**Incremental learning reduces necessity of corrective interactions**. For many tasks, i is notably higher and n lower when comparing systems with learning to systems without learning, indicating that the feedback from earlier (failed) attempts is effectively utilized to improve following executions of the same task. This effect is also confirmed by Supplementary Figures 1, 2. Although for GPT-4 on seen instructions, performance is already on a high level and corrections are rarely necessary, and the numbers strongly support that incremental learning reduces interactions for unseen instructions, as well as for GPT-3.5 on all instructions. Thus, our method for incremental learning is especially useful for “hard” tasks with respect to the predefined examples and general capabilities of the used model.

**Incremental learning improves the in-task success rate**. Our systems with incremental learning also have higher s than those without learning. The reason is that our incremental learning method reflects on the erroneous behavior and generates a new sample for in-context learning that demonstrates the desired behavior. With such nearly identical demonstration, the generalization to new situations is much better, thus causing fewer errors that cannot be corrected through interaction.

**Incremental learning generalizes to new tasks**. Qualitatively, we observed several cases where a correction for one task is useful for another task as well. For instance, GPT-3.5 initially interprets “the corner” as some position like (0.1,0.9) . When instructing to “put it right into the corner without any margin,” the behavior of using full numbers, e.g., (0,1) , transfers to subsequent different tasks that also involve corners. Quantitatively, this effect is entangled with the previous points in higher s and i , especially for the later unseen tasks. For a further investigation, see Supplementary Appendix C.

**Demonstration retrieval improves performance**. For both LLMs, our system with retrieval outperforms the system that always uses all samples. This is especially true for GPT-3.5 as the system without retrieval accumulated too many interaction examples in its memory in the final experiments, thus leading to immediate failure due to exceeding the LLM’s token limit. Although this is not the case for GPT-4 with its much larger context length, the performance of the system with retrieval is still better. We hypothesize that this is due to too many irrelevant samples distracting the LLM.

**Better LLMs lead to better performance**. This can be clearly seen when comparing the numbers for GPT-4 and GPT-3.5. Nonetheless, we emphasize that GPT-3.5’s performance as $L_{interact}$ is still reasonably well, while it is faster and a factor of 10 times cheaper. Specifically, the total cost to perform the experiments in Table 1 was $ 245.6 for GPT-4 vs. $ 19.8 for GPT-3.5 (which includes the use of GPT-4 for $L_{improve}$ ). Our method of incremental learning can thus be seen as a knowledge distillation method, with GPT-4 as the expensive teacher model $L_{improve}$ generating task-specific new prompts for the cheaper GPT-3.5 to improve its future behavior as $L_{interact}$ .

**Comparison with HELPER and Dynamic CaP**. As a key difference to our method, HELPER learns from successful trials by storing them as an example, while our method only inspects erroneous experiences and then stores improved versions thereof. The experimental results show that this strategy is more effective, leading to higher s,i and lower n . Furthermore, HELPER cannot see its own previously generated code when responding to errors or feedback, in contrast to our method, which utilizes the interactive Python console prompting for this purpose. Thus, HELPER cannot handle feedback such as “slightly more to the left” effectively.

Dynamic CaP improves performance over plain CaP, but it cannot compete with HELPER or our method. This confirms that our method of interactive Python console prompting is more effective than producing all code to solve the task at once. Furthermore, we can observe that learning from successful trials helps with seen instructions by reinforcing correct behavior, but it does not transfer to unseen instructions. Note that this observation also applies to HELPER, but mainly to i , since HELPER can better respond to execution errors and user feedback than CaP. We conclude that our proposed method to learn from erroneous interactions is more effective than reinforcing successful behavior only.

**Further results**. Supplementary Appendix C presents two additional experiments: first, we investigate the effect of k by setting k=4 (instead of 16), showing that lower k comes with a higher n and lower i , as potentially relevant demonstrations might not be retrieved, thus requiring another user interaction. Second, we change the behavior of $F_{learn}$ to simply save the current interaction in M , skipping $L_{improve}$ . This hurts the performance as the erroneous behavior from previous trials is often repeated, despite the prompt containing improvement instructions from earlier interactions.

## 5 Real-world demonstration

To demonstrate the utility of our proposed prompt-based incremental learning technique, we perform experiments on the real-world humanoid robot ARMAR-6 (Asfour et al., 2018). We first provide challenging commands which the LLM initially solves incompletely or wrongly. Then, the human interactively provides feedback and tells the robot how to improve. Afterward, we not only provide the same command again to check for improved behavior but, in order to study generalization, also try similar commands that initially (i.e., before learning) led to similar mistakes. Details on the implementation of these experiments, especially on the API exposed to the LLM, can be found in Supplementary Appendix D. The system is connected to a memory-centric cognitive robot architecture where the memory mediates between high-level components and low-level abilities (see Figure 4). Specifically, the API provided to the LLM allows querying the robot’s memory with functions to list all objects and location names (opt. with a given affordance), query sub-symbolic coordinates of objects or locations, or retrieve state information about specific objects. The robot’s memory is filled beforehand by the robot’s perception and cognition components. In our experiments, we use a mixture of predefined prior knowledge (e.g., about static objects in the scene) and online perception (e.g., object pose-detection, self-localization). Furthermore, the API allows invoking registered skills, behaviors, and movements of the robot, such as grasping, navigation, object placement, or handing objects to a human. However, we do not focus on scenarios where the involved skills themselves fail; rather, we focus on high-level semantic problems. Please refer to Supplementary Appendix D for further details.

We present three scenarios: *improving plans* to demonstrate complex improvement of suboptimal or unintended performance, *learning user preferences* to show how to adapt to non-generic task constraints, and *adapting low-level parameters* to demonstrate that our system can learn from vague user instructions. Demonstration videos can be found at https://lbaermann.github.io/interactive-incremental-robot-behavior-learning/.

### 5.1 Improving plans

In this scenario, we tell the robot that we want juice. The prior knowledge contains some similar interaction examples, picking up a single object and handing it over to the human. Thus, the task of bringing the juice is executed successfully. However, since the user needs a cup to drink, we further instruct the robot “can you also bring me a cup?,” which causes the robot to additionally hand over a cup. Afterward, we ask the robot to improve this behavior using “Thanks, but next time, can you do this both at once? You have two hands.” $L_{improve}$ generates an improved interaction example, as shown on the right of Listing 1 (simplified, cf. Supplementary Appendix E1).

Afterward, when given the same initial command again, the robot uses bimanual behavior to hand over both the juice and cup. Furthermore, the learned bimanuality generalizes to “can you bring something to drink to the table?” which does not use handover but places both objects on the table. Unfortunately, a further test with “can I have some milk, please?” shows the unimanual behavior again, so we again have to ask for a cup and trigger incremental learning. In the next session, we ask “hey, can you serve some drink?,” which correctly generalizes the behavior to use both hands to pick up a different drink and cup, but it misinterprets “serve” as performing a handover instead of putting it on the table. However, we can successfully trigger learning again by teaching “when I say serve, I mean that you should put it on the table,” and subsequent requests do behave as intended.

We conclude that our interactive, incremental learning system can flexibly generate complex behavior from concise improvement instructions. However, it is still challenging to robustly generalize from a single instruction to all the cases a human might have intended, as shown by the milk example, where a second correction was necessary for successful generalization. Improving this generalization capability should be a focus of future work.

### 5.2 Learning user preferences

As shown in Figure 1, in this scenario, we ask the robot to assist with cleaning the top of the fridge. The memory M contains predefined comparable examples for cleaning the table and kitchen counter, which guide the LLM to only handing over the sponge to the human. However, since the top of the fridge is higher than the table or the kitchen counter, we require a ladder to reach it, so we additionally ask for it (GPT-4 did, in contrast to GPT-3.5, proactively ask whether it should also bring the ladder). The robot then successfully places the ladder in front of the fridge. Eventually, we instruct the robot to always bring the ladder when working on high surfaces. The generated improved interaction example correctly brings the ladder after the sponge, without any further request (details in Supplementary Appendix E2). Afterward, when we perform a similar request (e.g., “clean on top of the dishwasher”), the robot brings both the sponge and the ladder successfully, while for lower surfaces (e.g., kitchen counter), the robot still brings only the sponge. The behavior also transfers to different tasks than cleaning, e.g., the robot brings the cereals and the ladder on “can you get me the cereals? I want to put it in the topmost shelf,” while it does not bring the ladder when tasked with “I want to put the cereals in the shelf.”

In summary, this example demonstrates that our method can be used to learn task constraints or preferences that a user specifies, and this knowledge can be generalized to similar situations.

### 5.3 Adapting low-level parameters

In this scenario, we ask the robot to bring some object from the table to the workbench (details in Supplementary Appendix E3). Subsequently, we say “remember that the route from the table to the bench is safe, you can go faster.” $F_{learn}$ correctly generates a sample that adapts the numeric speed factor of the move_to function on that route. However, if we test the same task afterward, $L_{interact}$ still uses the default speed. Annoyed by that, we shout “you forgot that I told you to go faster from the table to the workbench. When moving on that route, you should go faster!,” triggering another learning process, generating another correct sample, including an explicit comment, as shown in Listing 2.

Listing 2 When asked to move faster on a specific route, $L_{improve}$ generates an example including an explicit comment stating the user’s preference.

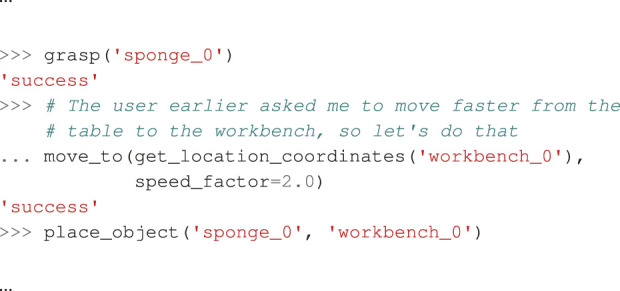

Proceeding requests now behave correctly and increase the speed from the table to the workbench. However, an adversarial test shows that $L_{interact}$ now dangerously uses increased speed from another location to the workbench as well, while routes to different places still correctly use the default speed.

To conclude, our system can successfully learn to adapt low-level API parameters as requested by a user, but ensuring that the LLM applies learned knowledge in the intended context only is not fully solved yet.

## 6 Conclusion and discussion

We present a system for integrating an LLM as the central part of high-level orchestration of a robot’s behavior in a closed interaction loop. Memorizing interaction examples from experience and retrieving them based on the similarity to the current user request allows for dynamic construction of prompts and enables the robot to incrementally learn from mistakes by extending its episodic memory with interactively improved code snippets. We describe our implementation of the system in the robot software framework ArmarX (Vahrenkamp et al., 2015) and on the humanoid robot ARMAR-6 (Asfour et al., 2018). The usefulness of our approach is evaluated both quantitatively on the tasks from Code as Policies (Ahn et al., 2022) and qualitatively on a humanoid robot in the real world.

Although the proposed method, in particular the incremental prompt learning strategy, shows promising results, there are still many open questions for real-world deployment. First of all, the performance of LLMs is quite sensitive to wording in the prompt, thus sometimes leading to unpredictable behavior despite only slight variations in the input (e.g., adding “please” in the user command). This might be solved with more advanced models in the future as we did observe this issue more often with GPT-3.5 than with GPT-4. Investigating the effect and performance of example retrieval in dynamic prompt construction might also contribute to improving robustness. Furthermore, our incremental prompt learning strategy should be expanded to involve additional human feedback before saving (potentially wrong) interaction examples to the episodic memory. However, this is challenging to accomplish if the user is not familiar with robotics or programming languages. One possible approach would be to verbalize the improved interaction example using an LLM, present it to the user, and ask for confirmation. Similarly, the improved code could first be executed in a simulation environment to check its validity before saving it in the memory of interaction examples. Both approaches have some open challenges, such as ensuring correctness of the verbalization or accuracy of the simulation as there will be a large sim-to-real gap for the type of behaviors considered in our paper. To rigorously evaluate our incremental learning method in the real world, future work may want to incorporate a user study with non-technical participants. Further work should also focus on abstraction of similar behavior and forgetting of irrelevant learned behavior. Although our system is limited by the APIs exposed to the LLM, it could be combined with complementary approaches (Parakh et al., 2023) to support learning of new low-level skills, which would then be exposed through new functions added to the API. Furthermore, designing an API that enables robust yet flexible interactions is a challenge that should be considered in future work. In particular, providing the LLM access to sub-symbolic parameters (such as positions to navigate to) enables fine-grained user corrections (“move a little more to the left”), but it can significantly harden the task for the LLM and entails many more failure cases. Moreover, although we provide the LLM with access to perception functions and examples of how to use them, it sometimes comes up with non-grounded behavior (e.g., referring to non-existing objects or locations). This may be improved by adding further levels of feedback to the LLM or using strategies like Grounded Decoding (Huang et al., 2023). Finally, our system inherits biases and other flaws from its LLM (Bender et al., 2021), which may lead to problematic utterances and behaviors. In future work, we will try to address some of these challenging questions to further push the boundaries of natural, real-world interactions with humanoid robots.

## Data Availability

The raw data supporting the conclusions of this article will be made available by the authors, without undue reservation.

## References

[B1] Ahmed A. Azab S. Abdelhamid Y. (2023). “Source-code generation using deep learning: a survey,” in Progress in artificial intelligence (Springer Nature Switzerland), 14116, 467–482. 10.1007/978-3-031-49011-8_37

[B2] Ahn M. Brohan A. Brown N. Chebotar Y. Cortes O. David B. (2022). Do as i can, not as i say: grounding language in robotic affordances. Annu. Conf. Rob. Learn.

[B3] Asfour T. Kaul L. Wächter M. Ottenhaus S. Weiner P. Rader S. (2018). “ARMAR-6: a collaborative humanoid robot for industrial environments,” in IEEE-RAS International Conference on Humanoid Robots, Beijing, China, November 06–09, 2018 (IEEE), 447–454.

[B4] Bao J. Hong Z. Tang H. Cheng Y. Jia Y. Xi N. (2016). “Teach robots understanding new object types and attributes through natural language instructions,” in IEEE International Conference on Sensing Technology, Nanjing, China, November 11–13, 2016 (IEEE), 1–6.

[B5] Bender E. M. Gebru T. McMillan-Major A. Shmitchell S. (2021). “On the dangers of stochastic parrots: can language models be too big?,” in 2021 ACM Conference on Fairness, Accountability, and Transparency, Virtual Event Canada, March 3–10, 2021, 610–623.

[B6] Birr T. Pohl C. Younes A. Asfour T. (2024). “Autogpt+p: affordance-based task planning using large language models,” in Proceedings of robotics: science and systems. Delft, Netherlands. 10.18653/v1/2022.emnlp-main.644

[B7] Brown T. Mann B. Ryder N. Subbiah M. Kaplan J. D. Dhariwal P. (2020). Language models are few-shot learners. Int. Conf. Neural Inf. Process. Syst. 33, 1877–1901. 10.5555/3495724.349588

[B8] Chen M. Tworek J. Jun H. Yuan Q. Pinto H. P. d. O. Kaplan J. (2021). Evaluating large language models trained on code. arXiv:2107.03374

[B9] Dalvi Mishra B. Tafjord O. Clark P. (2022). Towards teachable reasoning systems: using a dynamic memory of user feedback for continual system improvement. Conf. Emp. Meth. Nat. Lang. Proc., 9465–9480. 10.18653/v1/2022.emnlp-main.644

[B10] Dehaerne E. Dey B. Halder S. De Gendt S. Meert W. (2022). Code generation using machine learning: a systematic review. IEEE Access 10, 82434–82455. 10.1109/ACCESS.2022.3196347

[B11] Fasola J. Matarić M. J. (2013). Using semantic fields to model dynamic spatial relations in a robot architecture for natural language instruction of service robots. IEEE/RSJ Int. Conf. Intel. Rob. Syst. 32, 143–150. 10.1109/iros.2013.6696345

[B12] Forbes M. Rao R. Zettlemoyer L. Cakmak M. (2015). “Robot programming by demonstration with situated spatial language understanding,” in IEEE International Conference on Robotics and Automation, Seattle, WA, May 26–30, 2015 (IEEE).

[B13] Google (2023). Code-chat (Google VertexAI). Available at: https://cloud.google.com/vertex-ai/generative-ai/docs/model-reference/code-chat (Accessed April 11, 2024).

[B14] Guadarrama S. Riano L. Golland D. Göhring D. Jia Y. Klein D. (2013). Grounding spatial relations for human-robot interaction. IEEE/RSJ Int. Conf. Intel. Rob. Syst. 21, 1640–1647. 10.1109/iros.2013.6696569

[B15] Haluptzok P. Bowers M. Kalai A. T. (2023). “Language models can teach themselves to program better,” in International Conference on Learning Representations. Kigali Rwanda (Accessed May 1–5, 2023).

[B16] Huang W. Abbeel P. Pathak D. Mordatch I. (2022a). Language models as zero-shot planners: extracting actionable knowledge for embodied agents. Int. Conf. Mach. Learn. 162, 9118–9147.

[B17] Huang W. Xia F. Shah D. Driess D. Zeng A. Lu Y. (2023). Grounded decoding: guiding text generation with grounded models for robot control. arXiv:2303.00855

[B18] Huang W. Xia F. Xiao T. Chan H. Liang J. Florence P. (2022b). “Inner monologue: embodied reasoning through planning with language models,” in Annual Conference on Robot Learning. Auckland, New Zealand. (Accessed December 14–18, 2022).

[B19] Kartmann R. Asfour T. (2023). Interactive and incremental learning of spatial object relations from human demonstrations. Front. Robotics AI 10, 1151303. 10.3389/frobt.2023.1151303 PMC10232811 37275214

[B20] Krüger N. Geib C. Piater J. Petrick R. Steedman M. Wörgötter F. (2011). Object–Action Complexes: grounded abstractions of sensory–motor processes. Rob. Auton. Sys. 59, 740–757. 10.1016/j.robot.2011.05.009

[B21] Lahiri S. K. Fakhoury S. Naik A. Sakkas G. Chakraborty S. Musuvathi M. (2023). Interactive code generation via test-driven user-intent formalization. arXiv:2208.05950

[B22] Le H. Wang Y. Gotmare A. D. Savarese S. Hoi S. C. H. (2022). CodeRL: mastering code generation through pretrained models and deep reinforcement learning. Int. Conf. Neural Inf. Process. Syst. 35, 21314–21328. 10.5555/3600270.3601819

[B23] Liang J. Huang W. Xia F. Xu P. Hausman K. Ichter B. (2023). “Code as policies: language model programs for embodied control,” in IEEE International Conference on Robotics and Automation, London, United Kingdom, May 29–June 02, 2023 (IEEE), 9493–9500.

[B24] Liu J. Shen D. Zhang Y. Dolan B. Carin L. Chen W. (2022). “What makes good in-context examples for GPT-3?,” in Proceedings of Deep Learning Inside Out: The 3rd Workshop on Knowledge Extraction and Integration for Deep Learning Architectures, Dublin, Ireland (Association for Computational Linguistics), 100–114. 10.18653/v1/2022.deelio-1.10

[B25] Liu X. Yu H. Zhang H. Xu Y. Lei X. Lai H. (2024). “AgentBench: evaluating LLMs as agents,” in International Conference on Learning Representations Vienna Austria (Accessed May 7–11, 2024).

[B26] Luo M. Xu X. Dai Z. Pasupat P. Kazemi M. Baral C. (2023). Dr.ICL: demonstration-retrieved in-context learning. arXiv:2305.14128

[B27] Madaan A. Tandon N. Clark P. Yang Y. (2022a). Memory-assisted prompt editing to improve GPT-3 after deployment. Conf. Emp. Meth. Nat. Lang. Proc., 2833–2861. 10.18653/v1/2022.emnlp-main.183

[B28] Madaan A. Zhou S. Alon U. Yang Y. Neubig G. (2022b). Language models of code are few-shot commonsense learners. Conf. Emp. Meth. Nat. Lang. Proc., 1384–1403. 10.18653/v1/2022.emnlp-main.90

[B29] Mialon G. Dessi R. Lomeli M. Nalmpantis C. Pasunuru R. Raileanu R. (2023). Augmented language models: a survey. Trans. Mach. Learn. Res.

[B30] Misra D. K. Sung J. Lee K. Saxena A. (2016). Tell me Dave: context-sensitive grounding of natural language to manipulation instructions. Int. J. Rob. Res. 35, 281–300. 10.1177/0278364915602060

[B31] Mohan S. Laird J. (2014). Learning goal-oriented hierarchical tasks from situated interactive instruction. AAAI 28. 10.1609/aaai.v28i1.8756

[B32] Nicolescu M. Arnold N. Blankenburg J. Feil-Seifer D. Banisetty S. B. Nicolescu M. (2019). “Learning of complex-structured tasks from verbal instruction,” in IEEE-RAS 19th International Conference on Humanoid Robots (Humanoids), Toronto, ON, October 15–17, 2019 (IEEE), 770–777.

[B33] Nijkamp E. Pang B. Hayashi H. Tu L. Wang H. Zhou Y. (2023). “CodeGen: an open large language model for code with multi-turn program synthesis,” in International Conference on Learning Representations, Kigali, Rwanda, May 1–5, 2023.

[B34] OpenAI (2023a). ChatGPT. Available at: https://openai.com/blog/chatgpt/ (Accessed July 03, 2023).

[B35] OpenAI (2023b). GPT-4 technical report. arXiv:2303.08774.

[B36] Ouyang L. Wu J. Jiang X. Almeida D. Wainwright C. L. Mishkin P. (2022). Training language models to follow instructions with human feedback. Int. Conf. Neural Inf. Process. Syst. 35, 27730–27744. 10.5555/3600270.3602281

[B37] Parakh M. Fong A. Simeonov A. Gupta A. Chen T. Agrawal P. (2023). Lifelong robot learning with human assisted language planners. Work. learn. Eff. Abstr. Plan. CoRL.

[B38] Parisi A. Zhao Y. Fiedel N. (2022). TALM: tool augmented language models. arXiv:2205.12255

[B39] Peller-Konrad F. Kartmann R. Dreher C. R. G. Meixner A. Reister F. Grotz M. (2023). A memory system of a robot cognitive architecture and its implementation in ArmarX. Rob. Auton. Sys. 164, 104415. 10.1016/j.robot.2023.104415

[B40] Pramanick P. Barua H. B. Sarkar C. (2020). DeComplex: task planning from complex natural instructions by a collocating robot. IEEE/RSJ International Conference on Intelligent Robots and Systems (IROS), Las Vegas, NV, October 24, 2020–January 24, 2021 (IEEE), 8. 10.1109/IROS45743.2020.9341289

[B41] Qin Y. Hu S. Lin Y. Chen W. Ding N. Cui G. (2023). Tool learning with foundation models. arXiv:2304.08354

[B42] Reimers N. Gurevych I. (2019). Sentence-BERT: sentence embeddings using siamese BERT-networks. Conf. Emp. Meth. Nat. Lang. Proc., 3982–3992. 10.18653/v1/D19-1410

[B43] Ren A. Z. Dixit A. Bodrova A. Singh S. Tu S. Brown N. (2023). “Robots that ask for help: uncertainty alignment for large language model planners,” in Conference on Robot Learning (CoRL), Atlanta, USA, November 6–9, 2023.

[B44] Sarch G. Wu Y. Tarr M. Fragkiadaki K. (2023). Open-ended instructable embodied agents with memory-augmented large language models. Conf. Emp. Meth. Nat. Lang. Proc., 3468–3500. 10.18653/v1/2023.findings-emnlp.226

[B45] Shridhar M. Mittal D. Hsu D. (2020). INGRESS: interactive visual grounding of referring expressions. Int. J. Rob. Res. 39, 217–232. 10.1177/0278364919897133

[B46] Singh I. Blukis V. Mousavian A. Goyal A. Xu D. Tremblay J. (2023). “ProgPrompt: generating situated robot task plans using large language models,” in International Conference on Robotics and Automation (ICRA), London, United Kingdom, May 29–June 2, 2023, 11523–11530.

[B47] Skreta M. Yoshikawa N. Arellano-Rubach S. Ji Z. Kristensen L. B. Darvish K. (2023). Errors are useful prompts: instruction guided task programming with verifier-assisted iterative prompting. arXiv:2303.14100

[B48] Song C. H. Wu J. Washington C. Sadler B. M. Chao W.-L. Su Y. (2023). “LLM-planner: few-shot grounded planning for embodied agents with large language models,” in International Conference on Computer Vision (ICCV), Paris, France, October 01–06, 2023 (IEEE), 2998–3009.

[B49] Tellex S. Gopalan N. Kress-Gazit H. Matuszek C. (2020). Robots that use language: a survey. Annu. Rev. Control Rob. Auton. Sys. 3, 25–55. 10.1146/annurev-control-101119-071628

[B50] Tellex S. Kollar T. Dickerson S. Walter M. R. Banerjee A. G. Teller S. (2011). Understanding natural language commands for robotic navigation and mobile manipulation. AAAI 25 (1), 1507–1514. 10.1609/aaai.v25i1.7979

[B51] Touvron H. Lavril T. Izacard G. Martinet X. Lachaux M.-A. Lacroix T. (2023). LLaMA: open and efficient foundation language models. arXiv:2302.13971

[B52] Vahrenkamp N. Wächter M. Kröhnert M. Welke K. Asfour T. (2015). The robot software framework ArmarX. it - Inf. Technol. 57, 99–111. 10.1515/itit-2014-1066

[B53] Vemprala S. Bonatti R. Bucker A. Kapoor A. (2023). ChatGPT for robotics: design principles and model abilities. Available at: https://www.microsoft.com/en-us/research/publication/chatgpt-for-robotics-design-principles-and-model-abilities/ (Accessed February 22, 2023).

[B54] Wake N. Kanehira A. Sasabuchi K. Takamatsu J. Ikeuchi K. (2023). ChatGPT empowered long-step robot control in various environments: a case application. IEEE Access 11, 95060–95078. 10.1109/access.2023.3310935

[B55] Walter M. Hemachandra S. Homberg B. Tellex S. Teller S. (2013). “Learning semantic maps from natural language descriptions,” in Proceedings of the 2013 Robotics: Science and Systems IX Conference, Berlin, Germany, June 24–28, 2013.

[B56] Wang G. Xie Y. Jiang Y. Mandlekar A. Xiao C. Zhu Y. (2024). Voyager: an open-ended embodied agent with large language models. Trans. Mach. Learn. Res.

[B57] Wang J. Chen Y. (2023). A review on code generation with LLMs: application and evaluation. Int. Conf. Med. Art. Intel. 32, 284–289. 10.1109/MedAI59581.2023.00044

[B58] Wang X. Wang Z. Liu J. Chen Y. Yuan L. Peng H. (2024). “MINT: evaluating LLMs in multi-turn interaction with tools and language feedback,” in International Conference on Learning Representations, Vienna Austria, May 7–11, 2024.

[B59] Wang Z. Zhang G. Yang K. Shi N. Zhou W. Hao S. (2023). Interactive natural language processing. arXiv:2305.13246

[B60] Wei J. Wang X. Schuurmans D. Bosma M. ichter b. Xia F. (2022). “Chain-of-thought prompting elicits reasoning in large language models,” in International Conference on Neural Information Processing Systems, New Orleans, United States, November 28–December 09, 2022.

[B61] Weigelt S. Steurer V. Hey T. Tichy W. F. (2020). “Programming in natural language with fuSE: synthesizing methods from spoken utterances using deep natural language understanding,” in Proceedings of the 58th annual meeting of the association for computational linguistics (Association for Computational Linguistics), 4280–4295.

[B62] Wu J. Antonova R. Kan A. Lepert M. Zeng A. Song S. (2023). TidyBot: personalized robot assistance with large language models. Auton. Robots 47, 1087–1102. arXiv:2305.05658. 10.1007/s10514-023-10139-z

[B63] Yang J. Prabhakar A. Narasimhan K. Yao S. (2023). “InterCode: standardizing and benchmarking interactive coding with execution feedback,” in Proceedings of the 37th International Conference on Neural Information Processing System, New Orleans, LA, December 10–16, 2023 (Curran Associates Inc.), 23826–23854.36

[B64] Yang K. Liu J. Wu J. Yang C. Fung Y. Li S. (2024). “If LLM is the wizard, then code is the wand: a survey on how code empowers large language models to serve as intelligent agents,” in ICLR 2024 Workshop on LLM Agents, Vienna, Austria, May 11, 2024.

[B65] Yao S. Zhao J. Yu D. Du N. Shafran I. Narasimhan K. R. (2023). “ReAct: synergizing reasoning and acting in language models,” in International Conference on Learning Representations, Kigali, Rwanda, May 1–5, 2023.

[B66] Ye J. Wu Z. Feng J. Yu T. Kong L. (2023). Compositional exemplars for in-context learning. arXiv:2302.05698

[B67] Zeng A. Attarian M. ichter b. Choromanski K. M. Wong A. Welker S. (2023). “Socratic models: composing zero-shot multimodal reasoning with language,” in International Conference on Learning Representations, Kigali, Rwanda, May 1–5, 2023.

[B68] Zha L. Cui Y. Lin L.-H. Kwon M. Arenas M. G. Zeng A. (2023). “Distilling and retrieving generalizable knowledge for robot manipulation via language corrections,” in Work. Lang. Robot learn., CoRL.

[B69] Zheng Z. Ning K. Wang Y. Zhang J. Zheng D. Ye M. (2024). A survey of large language models for code: evolution, benchmarking, and future trends. arXiv:2311.10372

