# Supplementary material for: Incremental learning of humanoid robot behavior from natural interaction and large language models
Source: Front Robot AI. 2024 Oct 10;11:1455375. doi: 10.3389/frobt.2024.1455375 (PMC11499633; doi:10.3389/frobt.2024.1455375)
Supplement: Supplementary file 1 [file DataSheet1.pdf]

Supplementary Material

A CODE AS POLICIES EVALUATION – SETUP DETAILS

All evaluated systems generate code that utilizes the same API as in Liang et al. (2023). Specifically, the LLM can access the following functions:

- `denormalize_xy(pos_normalized)`
  Takes in a normalized position (coordinates between 0 and 1) and returns the denormalized position that can be used to execute a grasping or placing operation.
- `get_obj_names()`
  Returns the objects in the scene as a list of strings.
- `get_obj_pos(obj_name)`
  Returns the (denormalized) position of the object in the scene. If the object does not exist, it throws a `SemanticHintError` hinting at using one of the existing objects.
- `put_first_on_second(obj_name_1, target_name_or_xy_pos)`
  Executes pick and place. Both arguments can either be object names or position values.

The perception functions access the simulation state, so they deliver perfect results. The scripted motion plans of `put_first_on_second` taken from Liang et al. (2023) are not perfect as they do not take possible collisions into account, thus sometimes dropping blocks.

Our method can additionally use the two built-in functions

- `wait_for_trigger()`
  Will wait for additional human input, and also performs automatic success detection to log the state of the experiment.
- `learn_from_interaction()`
  Triggers incremental learning as explained in Section 3.5.

One run of our experiments goes as follows:

1. Randomly generate a scene and pick attributes for the placeholders in the current instruction template (e.g., for the instruction "pick up the <*block*> and place it on the <*corner*>", pick $block$ = red and $corner$ = top left corner)
2. Pass the instruction to the system
3. Wait for the system to return (i.e. finish executing the generated code for CaP and invoking F_{wait} for our method). At this step, the run can fail with an error (i.e. uncatched exception) or timeout.
4. Run automatic success detection, log the result (either success or failure).
5. Present the result to the human observer and ask for further instruction.
6. If there is no further instruction, the run ends in the current state. Otherwise, repeat from 2.

All runs of all instructions are performed in sequence. This means that the simulation environment and the evaluated system are reset between each run, but the interaction examples memory is kept (in order to allow incremental learning).

HELPER implementation details The prompts provided by Sarch et al. (2023) are designed for the TEACh (Padmakumar et al., 2022) environment, so we adapt them to the CaP simulated tabletop evaluation scenario & API explained above. HELPER originally uses a vision-language model (VLM) to pick the most probable reason from common failure cases given the current camera image. Since these failure cases are highly specific for the TEACh environment, we replace this mechanism with feeding back execution errors as given by the simulation environment directly to HELPER's *Self-Reflection & Correction* prompt (i.e. not involving a VLM). For a fair comparison, we handle the same set of errors as for our method, while the non-caught errors fail the experiment immediately (e.g. SyntaxError).

Sarch et al. (2023) also propose to utilize user feedback on completion of the program. In their experiments, this feedback is generated programmatically by comparing the current and ground-truth goal state of the TEACh environment. In our experiments, we use the human observer to provide this feedback, which is then passed as a new command to the PLANNER. Note that the design of HELPER's prompting method does not allow the system to inspect its previously generated code when responding to errors or feedback, in contrast to our Python console prompting.

HELPER's few-shot memory is expanded with successful trials as indicated by the human supervisor. Specifically, if the simulation state is *success*, and the human does not provide further feedback, the most recent instruction and generated code are stored in the example memory. We initialize the few-shot example memory with the same examples used by CaP and our method, translated to the prompting style of HELPER. For a fair comparison, we also set $k = 16$ for top-k example retrieval of similar instructions. In contrast, for the database of failure handling examples (which is separate from the "normal" examples in HELPER), we follow HELPER and provide only $k = 3$ examples (and there is also no incremental learning). The code and prompts for evaluating HELPER can be found in our GitHub repository.

Dynamic CaP implementation details For a fair comparison of our method with CaP, we add a simple form of learning and top-k retrieval and call this method *Dynamic CaP*. Specifically, similar to HELPER and our method, we define a memory of few-shot samples, initialized with the ones from the original static prompt. Further, also following HELPER, we store the code transcript after successful episodes as a new sample in this memory. On every request, we fill the prompt with the top-k retrieved examples from memory, according to semantic similarity of the query. Note that this few-shot retrieval and storing mechanism also applies to the nested language model programs (LMP) used by CaP, "`parse_obj_name`" and "`parse_position`". We set k to the same value as used in the original static prompts, i.e. $k = 16$ for the main LMP, $k = 8$ for "`parse_obj_name`" and $k = 9$ for "`parse_position`".

B CODE AS POLICIES EVALUATION – INTERACTION DETAILS & EXAMPLES

For our experiments, human feedback was provided by one individual (the first author) for all runs, respecting the following guidelines:

- First provide high-level feedback on how to solve the task correctly, only if that fails multiple times or the initial condition is already destroyed, give the solution via natural language. Consider the example task "pick up the first block from the bottom and put it in the top right corner". The system might flip the coordinates and pick the wrong block. Appropriate feedback would be "you did not correctly identify the first block from the bottom. you picked the topmost block instead.". If such feedback does

not help multiple times or the initial condition is already destroyed (in the example, if the task was to put the block in the bottom right corner, another block would already be moved to the bottom, so correctly following the initial task to pick up the bottommost block would not result in picking the correct block), feedback like “it is the blue block I was asking for” would be valid.

- The feedback is independent of the code produced by each evaluated method, i.e. the human observes only the actions visible in the simulation rendering. Direct feedback on the code (“you flipped the coordinates in line 2”) is never provided.
- If there are simple execution errors (e.g. a block is dropped), the feedback is to state the problem and ask to repeat the action (“you dropped the red block, try that one again”).
- Feedback is always given exhaustively, trying to reach success. Therefore, experiment runs either end with success, error, or timeout (this especially happened when the simulated gripper tried to calculate inverse kinematics for unreachable points). The failure state (no error, but goal not reached) is only a temporary state, because it still allows for further feedback. The only exception is when the human observer identified that the world state makes it impossible to solve the task, which means further feedback would be useless, for instance when the robot dropped a task-relevant block in an unfortunate way so that it ends up outside of its action space.
- Incremental learning is triggered if the system first failed to solve a task, and then managed to do it correctly without the human providing the solution explicitly. For instance, when a task was solved only after the human revealed which block is the correct one, no request for incremental learning was given. Similarly, incremental learning was not requested when the feedback was only necessary due to execution errors such as dropping a block.

The remainder of this section lists some example interactions from the CaP evaluation to showcase the different type of feedback provided by human supervision. The human feedback can be found in the `'text'` property in the return value of the `wait_for_trigger()` calls (except for the first one in each interaction, which returns the initial command).

ours, gpt-4, UA/UI:

This is an example of the LLM performing some behavior in a principally correct way, but without considering the physical side effects (destroying the stack when moving all blocks at once). The human feedback first instructs how to do it correctly, and then asks to learn from that mistake.

```
>>> wait_for_trigger()
{'type': 'dialog', 'text': 'stack all the blocks on the bottom side'}
>>> bottom_side_pos = denormalize_xy([0.5, 0])
>>> block_names = [name for name in get_obj_names() if 'block' in name]
>>> for i in range(len(block_names) - 1):
...     put_first_on_second(block_names[i + 1], block_names[i])
>>> put_first_on_second(block_names[0], bottom_side_pos)
'success'
>>> wait_for_trigger()
{'type': 'dialog', 'text': 'by stacking them first and then moving, you destroyed the stack. '
                           'create the stack directly at the target location'}
>>> block_names = [name for name in get_obj_names() if 'block' in name]
>>> for i in range(len(block_names)):
...     put_first_on_second(block_names[i], bottom_side_pos)
>>> wait_for_trigger()
{'type': 'dialog', 'text': 'well done, learn that for next time'}
>>> learn_from_interaction()
```

The following two examples demonstrate how the LLM can adapt subsymbolic parameters given a vague user instruction.

```
>>> wait_for_trigger()
{'type': 'dialog', 'text': 'pick up the gray block and place it a lot to the right of the gray bowl'}
>>> get_obj_names()
['gray block', 'red block', 'gray bowl', 'cyan bowl']
>>> gray_bowl_pos = get_obj_pos('gray bowl')
>>> target_pos = gray_bowl_pos + [0.2, 0]  # move a lot to the right
>>> put_first_on_second('gray block', target_pos)
'success'
>>> wait_for_trigger()
{'type': 'dialog', 'text': 'a little further right, please'}
>>> target_pos = target_pos + [0.1, 0]  # move a little further to the right
>>> put_first_on_second('gray block', target_pos)
'success'
```

```
>>> wait_for_trigger()
{'type': 'dialog', 'text': 'pick up the gray block and place it a lot to the right of the purple bowl'}
>>> get_obj_names()
['gray block', 'cyan block', 'purple bowl', 'pink bowl', 'gray bowl', 'yellow bowl']
>>> purple_bowl_pos = get_obj_pos('purple bowl')
>>> target_pos = purple_bowl_pos + [1.0, 0]  # a lot to the right
>>> put_first_on_second('gray block', target_pos)

Point not in boundaries. Make sure to use denormalize_xy correctly.

>>> target_pos = denormalize_xy([1.0, purple_bowl_pos[1]])
>>> put_first_on_second('gray block', target_pos)

Point not in boundaries. Make sure to use denormalize_xy correctly.

>>> target_pos = purple_bowl_pos + [0.5, 0]  # a bit to the right
>>> put_first_on_second('gray block', target_pos)
'success'
>>> wait_for_trigger()
{'type': 'dialog', 'text': 'thats a bit too far, try half of the distance'}
>>> target_pos = purple_bowl_pos + [0.25, 0]  # half of the previous distance
>>> put_first_on_second('gray block', target_pos)
'success'
>>> wait_for_trigger()
{'type': 'dialog', 'text': 'well done, learn from your mistakes'}
```

CaP, gpt-4, UA/SI:

In this example, the LLM did generate an empty response on the first request. Given the clarification instruction, it performs the task correctly.

```
# put all the blocks in the bowls with matching colors.
objects = ['red block', 'orange block', 'yellow block', 'yellow bowl', 'orange bowl', 'red bowl']
# the yellow block in the yellow bowl etc.
objects = ['red block', 'orange block', 'yellow block', 'yellow bowl', 'orange bowl', 'red bowl']
put_first_on_second('yellow block', 'yellow bowl')
put_first_on_second('orange block', 'orange bowl')
put_first_on_second('red block', 'red bowl')
```

ours w/o learn, gpt-4, SA/SI:

This example shows that the behavior of the LLM occasionally does not use the correct reasoning, but arrives at the correct solution (after multiple human corrections) by chance. Our experiments solely evaluate the visible outcome. The human supervisor does not observe the code generated by the LLM.

```
>>> wait_for_trigger()
{'type': 'dialog', 'text': 'pick up the first block from the top and place it on the top side'}
>>> get_obj_names()
['green block', 'orange block', 'red block', 'brown bowl', 'purple bowl']
>>> top_side_pos = denormalize_xy([0.5, 1])
>>> put_first_on_second('green block', top_side_pos)
```

```
'success'
>>> wait_for_trigger()
{'type': 'dialog', 'text': 'no, that was the first block from the bottom.'}
>>> put_first_on_second('red block', top_side_pos)
'success'
>>> wait_for_trigger()
{'type': 'dialog', 'text': 'still not correct'}
>>> put_first_on_second('orange block', top_side_pos)
'success'
>>> wait_for_trigger()
```

ours w/o retrieval, gpt-4, SA/SI

This example demonstrates that the human feedback sometimes does not need to correct a misunderstanding, but solely provides environment feedback about the robot's actions having unintended side effects or not working as expected. For instance, here the robot did drop the stack of blocks while putting the last one on top.

```
>>> wait_for_trigger()
{'type': 'dialog', 'text': 'put the blocks in the green bowl'}
>>> get_obj_names()
['green block', 'gray block', 'red block', 'orange block', 'green bowl', 'brown bowl', 'purple bowl']
>>> put_first_on_second('green block', 'green bowl')
'success'
>>> put_first_on_second('gray block', 'green bowl')
'success'
>>> put_first_on_second('red block', 'green bowl')
'success'
>>> put_first_on_second('orange block', 'green bowl')
'success'
>>> wait_for_trigger()
{'type': 'dialog', 'text': 'you dropped them, try again'}
>>> put_first_on_second('green block', 'green bowl')
'success'
>>> put_first_on_second('gray block', 'green bowl')
'success'
>>> put_first_on_second('red block', 'green bowl')
'success'
>>> put_first_on_second('orange block', 'green bowl')
'success'
>>> wait_for_trigger()
{'type': 'dialog', 'text': 'the gray one is still missing'}
>>> put_first_on_second('gray block', 'green bowl')
'success'
>>> wait_for_trigger()
```

ours, gpt-3.5, SA/SI

This example nicely demonstrates that *(i)* `gpt-3.5` does much more errors initially, as it misunderstands top right corner here (`gpt-4` always picked up the correct coordinate system from the examples and never did that mistake), *(ii)* our method enables learning from mistakes by just mentioning the mistake, without providing further help on how to achieve the goal ("this is the bottom right corner, not the top right"), and *(iii)* the LLM can perform corrections of subsymbolic values given vague user instructions ("without a margin").

```
>>> wait_for_trigger()
{'type': 'dialog', 'text': 'put all the blocks on the top right corner'}
>>> block_names = [name for name in get_obj_names() if 'block' in name]
>>> block_positions = [get_obj_pos(name) for name in block_names]
>>> top_right_pos = denormalize_xy([0.9, 0.1])
>>> for block_name in block_names:
...     put_first_on_second(block_name, top_right_pos)
>>> wait_for_trigger()
{'type': 'dialog', 'text': 'this is the bottom right corner, not the top right'}
>>> block_names = [name for name in get_obj_names() if 'block' in name]
>>> block_positions = [get_obj_pos(name) for name in block_names]
>>> top_right_pos = denormalize_xy([0.9, 0.9])
```

```
>>> for block_name in block_names:
...     put_first_on_second(block_name, top_right_pos)
>>> wait_for_trigger()
{'type': 'dialog', 'text': 'good, but please put it right at the top right corner without margin'}
>>> block_names = [name for name in get_obj_names() if 'block' in name]
>>> block_positions = [get_obj_pos(name) for name in block_names]
>>> top_right_pos = denormalize_xy([1.0, 1.0])
>>> for block_name in block_names:
...     put_first_on_second(block_name, top_right_pos)
>>> wait_for_trigger()
{'type': 'dialog', 'text': 'great, learn from that for next time'}
>>> learn_from_interaction()
```

C CODE AS POLICIES EVALUATION – DETAILED RESULTS

Table S2 and S3 show detailed, per-instruction results for our experiments with `gpt-3.5` and `gpt-4`, respectively. The following paragraphs elaborate additional experiments:

Setting $k = 4$. To investigate the effect of the hyperparameter k defining the number of few-shot samples to select from $\mathcal{M}$, we performed another experiment with $k = 4$. Note that *ours* has $k = 16$ and *ours w/o learning* has $k = 64$. From the results for `gpt-3.5`, we can see that a lower k decreases i and increases n, as relevant examples might not be retrieved and thus more interaction is required. For unseen tasks, the lower amount of few-shot samples also decreases the overall success rate s, as more runs fail with an error.

In contrast, `gpt-4` is able to perform very well even for $k = 4$, significantly reducing the costs. While an initial drop in i can be seen for the first runs with seen instructions, incremental learning can level out this effect so that the systems performs similar or even better than our method with $k = 16$.

Ablating L_{improve}. In order to confirm the effect of L_{improve}, we perform another ablation experiment where F_{learn} simply stores the current interaction $\mathcal{H}_t$ in $\mathcal{M}$, instead of calling L_{improve} to generate $\mathcal{H}_t^*$. In theory, this kind of learning could still work, as $\mathcal{H}_t$ also includes the user feedback, thus allowing L_{interact} to use this feedback in future interactions. However, our results for `gpt-3.5` show that this strategy performs worse than with L_{improve}, while it still outperforms the system without learning. Especially, we observed that the system often just repeated the erroneous behavior from the saved example, instead of observing the feedback contained therein, thus requiring another user interaction. Note that for `gpt-3.5` ours w/o improve, we had to set $k = 12$ to avoid frequent errors due to reaching the token limit of the LLM, as the non-improved stored interactions are typically longer than the improved ones.

For `gpt-4`, there is no such problem with the context length, thus we kept $k = 16$ here. Due to the generally high performance, the positive effect of using L_{improve} is not that pronounced. For unseen instructions, the method even performs on par or slightly better than our system, as `gpt-4` is able to pick up (comparatively easy) improvement instructions from previous runs. However, we could still see that sometimes multiple corrections of the same mistake are necessary, since subsequent interactions first repeated the wrong behavior (as can be seen by lower i on SA SI).

Qualitatively, we investigated this effect further by comparing our system to that without L_{improve} on the real-world demonstration scenarios. These scenarios require significantly more complex behavior changes than the simulated evaluation. Thus, here we could clearly see that the system without L_{improve} cannot perform as well as our full system. Specifically, using the same instructions as in Section E.1, we were not able to achieve the bimanual transport of both the drink and cup, although we did instruct the system to learn that twice. Similarly, in the scenario detailed in Section E.2, the system never brought the ladder without asking. Instead, the system repeats the behavior from the learned sample, including the unnecessary interaction. For the scenario of Section E.3, the system without L_{improve} interestingly picks up the sped-up behavior after a single instruction for improvement, instead of two as for our full system. However, it did so without respecting the constraint of which route is safe, i.e. it simply uses increased speed everywhere. When instructed again to take care to only fasten up on the safe route, it does not use the increased speed at all anymore.

To sum up, we conclude that our method of using CoT prompting with L_{improve} to produce an improved interaction example is useful especially for weaker models (`gpt-3.5`) and harder tasks that involve complex behavioral adaptations. The simpler method of just storing the erroneous interaction including the

	ours		ours w/o learning	
	s	i	s	i
gpt-3.5	61.1	33.3	83.3	38.9
gpt-4	100	83.3	94.4	72.2

Table S1. Results of the dedicated experiment to investigate out-of-task generalization

user feedback works only for `gpt-4`, and only in case the instructions concern only local changes (such as changing a single parameter).

Numerical support for out-of-task generalization. During the experiments, we observed that improvements learned from earlier tasks can benefit later tasks, such as when `gpt-3.5` initially interprets "top left corner" as $(0.1, 0.9)$ instead of $(0, 1)$, but after correction and learning, corners are used correctly in unseen tasks as well. To further investigate this effect, we performed another small experiment: initialize our system with the memory content from after *unseen attributes, seen instructions*, and then evaluate on the unseen tasks. We repeat each unseen task three times without incremental learning, so that each run can still be considered "unseen". This is compared to the system that does not use incremental learning at all, i.e. the memory is initialized with the predefined prompts only.

The results are shown in Table S1. For `gpt-4`, the numbers support that our strategy for incremental learning generalizes to unseen tasks, improving both s and i. However, for `gpt-3.5`, we observe the opposite: The performance of the system initialized with the knowledge from previous execution of the seen tasks is worse than that of the system that has only the predefined interaction examples. We hypothesize that this is due to `gpt-3.5` easily overfitting to the prompt contents (that contain examples of the seen tasks, but not the unseen ones). Nevertheless, when not constrained to this specific evaluation setting, our system with incremental learning performed better on the unseen tasks, as it can learn about them on the fly (as shown by the numbers in Table S2).

Cost comparison When looking at Table S2 and S3, the costs of our method is 5 to 13 times higher than CaP or HELPER. The main cost driving factor is the scheme of repeated individual requests caused by the interactive Python console prompting. Specifically, the LLM typically generates solely one statement, this is executed, and this repeats. Each generation, however, involves a full prompt including API definition, few-shot examples and the current interaction. Thus, when using the OpenAI API, the cost of our method scales linearly with the number of console statements the LLM produces.

However, this cost could be significantly reduced if we had direct access to the LLM. This is due to the fact that the selection of few-shot examples only changes when there is new user input. Thus, instead of treating each request independently, the LLM could be used in an "incremental" mode by generating one statement, keeping the transformer hidden states in memory, force-decoding (i.e. programatically inserting) the return value of the execution of the generated statement, and then continuing to decode the next statement. That way, the computational cost of our method would be comparable to HELPER and CaP, which generate the full Python script at once.

Nonetheless, in comparison to HELPER and CaP, our prompting scheme has some token overhead. This is due to emulating a Python console, especially including the return value of each executed function. For instance, the length of the first prompt in the first run of our tabletop evaluation (before any learning happened, so the information in these prompts is equivalent) is 2682 tokens for our method, 1992 for HELPER, and 1570 for CaP. Furthermore, we have the special calls to F_{learn}, which incur additional

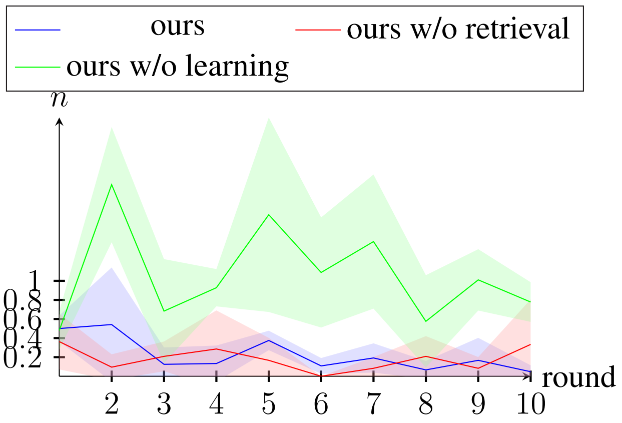

Figure S1a: Num interactions until success per round

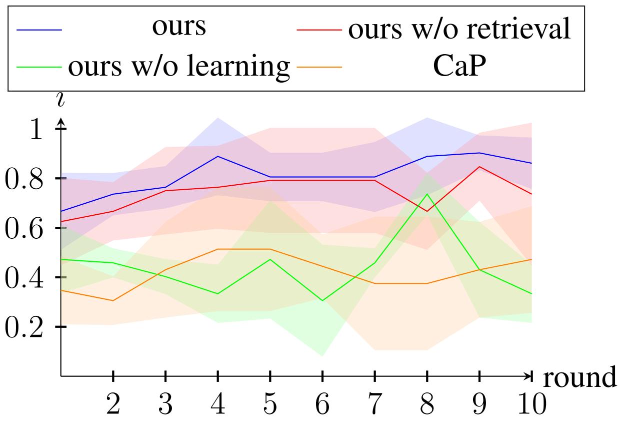

Figure S1b: initial success per round

Figure S1: results per round, gpt-3.5 experiments

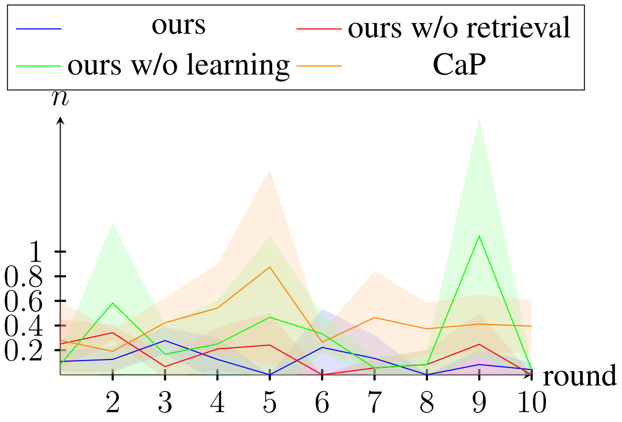

Figure S2a: Num interactions until success per round

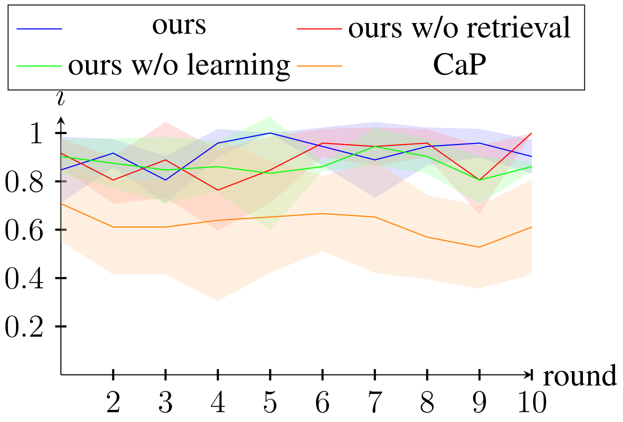

Figure S2b: initial success per round

Figure S2: results per round, gpt-4 experiments

computation budget. Thus, when using an "incremental" LLM mode as described above, the token cost of our method would be 2 to 3 times higher than CaP.

Performance analysis *per round*. Figs. S1 and S2 show initial success rate i and number of interactions until success n *per round*, which means that we aggregate the corresponding numbers over the x-th run of each instruction. For instance, the number for $x = 1$ in Fig. S1b is obtained by averaging the value of i from the `gpt-3.5` experiments in each first run over all instructions for each of the three blocks of Table S2 (SA/SI, UA/SI, UA/UI). Then, we take average and standard deviation over the three blocks to plot the curve and area in Fig. S1b.

The intention of these graphs is to show how incremental learning improves initial success rate and decreases the need for corrective interactions over time. When presented with a new instruction, it can be expected that some clarification is needed. However, after a few runs, the task should be clear, and the

system should perform well. Fig. S1 clearly confirms this for `gpt-3.5`. While there is some noise (e.g. due to the robot accidentally dropping some block and needing instruction to try again even in a later run), one can see a clear trend of n decreasing to 0 and i approaching 1 for our systems with incremental learning. In contrast, the systems without incremental learning have no such trend and show random variations only. For `gpt-4`, since the general performance is much better, the effect is not that emphasized in Fig. S2.

	ours			ours $k=4$			ours w/o retrieval			ours w/o improve			ours w/o learning			HELPER			Dynamic CaP			CaP		
	s	i	n	s	i	n	s	i	n	s	i	n	s	i	n	s	i	n	s	i	n	s	i	n
**Seen Attributes, Seen Instructions**																								
pick up the <block:1> and place it on the <block:2>	100	100	0.0	100	90	0.2	100	90	0.2	90	70	0.56	100	100	0.0	100	100	0.0	100	100	0.0	50	50	0.0
pick up the <block> and place it on the <bowl>	100	100	0.0	100	100	0.0	100	100	0.0	100	100	0.0	100	90	0.1	100	100	0.0	100	100	0.0	50	50	0.0
put all the blocks on the <corner/side>	100	90	0.2	100	90	0.2	100	80	0.3	100	90	0.1	100	50	1.3	100	100	0.0	40	40	0.0	90	80	0.11
put the blocks in the <bowl>	100	90	0.1	90	60	2.33	90	80	0.22	90	90	0.0	70	60	2.29	100	80	0.8	80	70	0.88	70	70	0.0
put all the blocks in the bowls with matching colors	100	90	0.1	100	40	1.4	70	70	0.0	100	100	0.0	20	20	0.0	100	100	0.0	90	80	0.11	90	90	0.0
pick up the block to the <direction> of the <bowl> and place it on the <corner/side>	80	50	0.75	90	30	1.33	90	70	0.44	70	30	1.43	100	0	2.8	50	10	1.0	0	0	–	10	10	0.0
pick up the block <distance> to the <bowl> and place it on the <corner/side>	100	100	0.0	100	100	0.0	100	90	0.2	100	100	0.0	90	10	1.0	100	30	0.7	50	50	0.0	70	70	0.0
pick up the <nth> block from the <direction> and place it on the <corner/side>	80	80	0.0	100	80	0.3	100	80	0.6	80	60	0.38	100	20	1.2	100	100	0.0	0	0	–	0	0	–
Total	95.0	87.5	0.14	97.5	73.8	0.72	93.8	82.5	0.25	91.2	80.0	0.31	85.0	43.8	1.09	93.8	77.5	0.31	57.5	55.0	0.16	53.8	52.5	0.02
Cost	1.7 $			3.14 $			2.7 $			1.2 $			1.8 $			0.3 $			0.3 $			0.3 $		
**Unseen Attributes, Seen Instructions**																								
pick up the <block:1> and place it on the <block:2>	100	90	0.2	100	100	0.0	100	100	0.0	80	50	0.5	100	90	0.1	100	100	0.0	100	100	0.0	40	40	0.0
pick up the <block> and place it on the <bowl>	100	100	0.0	100	100	0.0	100	100	0.0	100	100	0.0	100	100	0.0	100	100	0.0	100	100	0.0	90	90	0.0
put all the blocks on the <corner/side>	90	90	0.0	100	100	0.0	100	100	0.0	80	90	0.0	80	60	0.25	100	100	0.0	40	30	0.25	90	90	0.0
put the blocks in the <bowl>	100	80	1.1	90	80	1.44	100	80	0.3	90	50	0.78	70	30	1.71	90	80	0.22	90	80	0.56	90	80	0.33
put all the blocks in the bowls with matching colors	90	80	0.22	100	100	0.0	90	80	0.11	100	100	0.0	30	30	0.0	100	100	0.0	90	90	0.0	80	80	0.0
pick up the block to the <direction> of the <bowl> and place it on the <corner/side>	100	80	0.2	100	60	1.1	90	70	0.56	10	10	0.0	70	0	2.29	50	10	1.6	0	0	–	0	0	–
pick up the block <distance> to the <bowl> and place it on the <corner/side>	100	90	0.3	100	90	0.3	90	90	0.0	80	80	0.0	100	20	2.0	60	10	0.83	0	0	–	90	90	0.0
pick up the <nth> block from the <direction> and place it on the <corner/side>	100	80	0.6	80	80	0.0	100	90	0.2	30	30	0.0	90	30	1.22	100	70	0.4	100	60	0.6	0	0	–
Total	97.5	86.2	0.33	96.2	88.8	0.36	96.3	88.8	0.15	71.2	63.7	0.16	80.0	45.0	0.95	87.5	71.2	0.38	65.0	57.5	0.23	60.0	58.8	0.06
Cost	1.9 $			1.2 $			2.9 $			1.2 $			2.4 $			0.3 $			0.2 $			0.3 $		
**Unseen Attributes, Unseen Instructions**																								
put all the blocks in different corners	100	90	0.1	100	90	0.2	100	90	0.2	100	100	0.0	100	100	0.0	90	90	0.0	70	70	0.0	0	0	–
put the blocks in the bowls with mismatched colors	100	90	0.2	90	60	0.33	70	70	0.0	60	40	1.17	60	30	2.5	100	0	1.3	50	20	1.2	70	60	0.14
stack all the blocks on the <corner/side>	100	100	0.0	100	100	0.0	100	100	0.0	90	90	0.0	10	0	2.0	100	100	0.0	50	50	0.0	0	0	–
pick up the <block> and place it <magnitude> to the <direction> of the <bowl>	20	10	0.5	50	30	0.4	70	50	0.57	50	50	0.0	70	60	1.0	10	0	2.0	90	70	0.22	30	30	0.0
pick up the <block> and place it in the corner <distance> to the <bowl>	100	90	0.2	100	80	0.2	0	0	–	100	90	0.2	80	10	1.62	90	20	0.78	20	10	1.0	0	0	–
put all the blocks in a <line> line	90	40	0.67	20	20	0.0	0	0	–	0	0	–	80	60	0.62	90	90	0.0	0	0	–	0	0	–
Total	85.0	70.0	0.28	76.7	63.3	0.19	56.7	51.7	0.19	66.7	61.7	0.27	66.7	43.3	1.29	80.0	50.0	0.68	46.7	36.7	0.48	16.7	15.0	0.07
Cost	2.5 $			1.4 $			1.2 $			1.2 $			1.9 $			0.2 $			0.2 $			0.2 $		

Table S2. gpt-3.5 experiments (gpt-4 for L_{improve})

	ours			ours $k = 4$			ours w/o retrieval			ours w/o improve			ours w/o learning			HELPER			Dynamic CaP			CaP		
	s	i	n	s	i	n	s	i	n	s	i	n	s	i	n	s	i	n	s	i	n	s	i	n
**Seen Attributes, Seen Instructions**																								
pick up the <block:1> and place it on the <block:2>	100	100	0.0	100	100	0.0	100	100	0.0	100	100	0.0	100	100	0.0	100	100	0.0	100	100	0.0	100	100	0.0
pick up the <block> and place it on the <bowl>	100	100	0.0	100	100	0.0	100	100	0.0	100	100	0.0	100	100	0.0	100	100	0.0	100	100	0.0	100	100	0.0
put all the blocks on the <corner/side>	100	100	0.0	90	70	0.44	100	90	0.1	100	100	0.0	100	100	0.0	90	80	0.22	50	50	0.0	90	90	0.0
put the blocks in the <bowl>	100	90	0.2	100	90	0.1	100	80	0.3	100	80	0.6	100	80	1.6	100	90	0.1	100	90	0.3	90	90	0.0
put all the blocks in the bowls with matching colors	100	100	0.0	100	100	0.0	100	100	0.0	100	100	0.0	100	100	0.0	100	100	0.0	100	100	0.0	100	0	1.4
pick up the block to the <direction> of the <bowl> and place it on the <corner/side>	100	90	0.1	100	50	0.6	90	60	0.56	100	60	0.5	90	60	0.67	90	60	0.67	60	50	0.17	30	20	0.67
pick up the block <distance> to the <bowl> and place it on the <corner/side>	100	100	0.0	100	100	0.0	100	100	0.0	100	100	0.0	100	100	0.0	100	100	0.0	100	100	0.0	80	80	0.0
pick up the <nth> block from the <direction> and place it on the <corner/side>	100	100	0.0	100	100	0.0	90	90	0.0	100	100	0.0	100	80	0.7	100	70	0.7	100	100	0.0	90	90	0.0
Total	100	97.5	0.04	98.8	88.8	0.14	97.5	90.0	0.12	100	92.5	0.14	98.8	90.0	0.37	97.5	87.5	0.21	88.8	86.2	0.06	85.0	71.2	0.26
Cost	21.9 $			12 $			33 $			29.7 $			23.5 $			4.5 $			4.8 $			6 $		
**Unseen Attributes, Seen Instructions**																								
pick up the <block:1> and place it on the <block:2>	100	100	0.0	100	100	0.0	100	100	0.0	100	100	0.0	100	100	0.0	100	100	0.0	100	100	0.0	100	100	0.0
pick up the <block> and place it on the <bowl>	100	100	0.0	100	100	0.0	100	100	0.0	100	100	0.0	100	100	0.0	100	100	0.0	100	100	0.0	100	100	0.0
put all the blocks on the <corner/side>	100	100	0.0	100	100	0.0	100	100	0.0	100	100	0.0	100	100	0.0	100	100	0.0	100	100	0.0	100	100	0.0
put the blocks in the <bowl>	100	90	0.1	100	90	0.2	100	80	0.7	90	80	0.33	100	90	0.1	100	90	0.1	100	90	0.1	90	90	0.0
put all the blocks in the bowls with matching colors	100	100	0.0	100	100	0.0	100	100	0.0	100	100	0.0	100	100	0.0	100	100	0.0	100	100	0.0	100	0	2.2
pick up the block to the <direction> of the <bowl> and place it on the <corner/side>	100	60	0.9	100	90	0.1	90	80	0.22	100	80	0.2	90	50	0.67	100	60	0.7	80	60	0.5	80	60	0.62
pick up the block <distance> to the <bowl> and place it on the <corner/side>	100	90	0.1	100	100	0.0	100	100	0.0	100	100	0.0	100	100	0.0	100	100	0.0	100	100	0.0	100	100	0.0
pick up the <nth> block from the <direction> and place it on the <corner/side>	100	100	0.0	100	90	0.4	100	100	0.0	100	100	0.0	100	100	0.0	100	100	0.0	100	100	0.0	100	100	0.0
Total	100	92.5	0.14	100	96.3	0.09	98.8	95.0	0.12	98.8	95.0	0.07	98.8	92.5	0.1	100	93.8	0.1	97.5	93.8	0.07	96.2	81.2	0.35
Cost	45 $			12 $			23.7 $			31 $			19.4 $			4.6 $			6.1 $			6.3 $		
**Unseen Attributes, Unseen Instructions**																								
put all the blocks in different corners	100	100	0.0	100	100	0.0	100	100	0.0	100	100	0.0	100	100	0.0	100	100	0.0	100	100	0.0	0	0	–
put the blocks in the bowls with mismatched colors	100	80	0.3	100	80	0.8	100	70	0.6	100	90	0.2	100	70	0.3	100	80	0.4	80	60	0.75	80	0	2.25
stack all the blocks on the <corner/side>	100	90	0.1	100	100	0.0	100	100	0.0	100	90	0.1	100	100	0.0	100	100	0.0	80	40	1.5	100	100	0.0
pick up the <block> and place it <magnitude> to the <direction> of the <bowl>	60	50	0.17	80	70	0.12	50	50	0.0	80	70	0.12	50	30	0.8	50	40	0.6	80	40	0.88	40	20	0.5
pick up the <block> and place it in the corner <distance> to the <bowl>	100	100	0.0	100	90	0.2	100	100	0.0	100	100	0.0	100	100	0.0	100	100	0.0	0	0	–	0	0	–
put all the blocks in a <line> line	100	90	0.4	100	80	0.4	100	70	0.5	100	70	0.5	100	70	2.2	100	70	0.3	40	40	0.0	100	90	0.2
Total	93.3	85.0	0.16	96.7	86.7	0.25	91.7	81.7	0.18	96.7	86.7	0.15	91.7	78.3	0.55	91.7	81.7	0.22	63.3	46.7	0.62	53.3	35.0	0.74
Cost	20.2 $			10 $			16.9 $			19.7 $			20.7 $			4.4 $			7.2 $			9 $		

Table S3. gpt-4 experiments

D DEMONSTRATION IMPLEMENTATION DETAILS

All demonstration experiments were performed with `gpt-4-0613`. To give our system the ability to control a real-world humanoid robot, we define an API for the language model to use. The available functions are listed in the following. Each function has a name, type-annotated parameters and return value, and optionally a comment that is also shown to the LLM. Such comment is defined as a Python annotation directive directly at the function definition.

- `ask(question: str) -> str`
  Sends the given question to the TTS and blocks until the user responded, returning the utterance recognized by the ASR. It is up to the LLM to use either `ask(...)` or `say(...); wait_for_trigger()`.
- `bring_object_to(object_name: str, destination_object_name: str)` with comment: "full pick and place method. assumes that the object is already detected. Assure that using list_objects() before calling this"
  This method is a higher-level wrapper utilizing `move_to`, `grasp` and `place_object`. It moves to the source object's location if necessary, grasps it, moves to the location of the destination object, and places the object there. The LLM can use this function for easy pick-and-place tasks, however it does not give full control over the individual steps, e.g. in order to perform bimanual pick and place. We have predefined interaction examples involving `bring_object_to` as well as using the lower-level functions directly. A special case of this function is `bring_object_to('ladder', 'handover_to_human')`. This special case is demonstrated in a separate in-context example and causes the robot to bring and open the ladder at a predefined position (from where the human can take it). The implementation of `bring_object_to` branches on the provided object, invoking either the generic skill to bring some object from A to B, or the ladder-specific bimanual skill.
- `get_location_coordinates(location_name: str) -> np.ndarray`
  Returns the global coordinates of the specified location. If the location name does not exist in the robots semantic scene memory, a `SemanticHintError` is thrown, suggesting to call `list_locations()`.
- `get_obj_pos(obj_name: str) -> np.ndarray`
  Returns the 6D object pose of the specified object in global coordinates. If the object name does not exist in the robots semantic scene memory, a `SemanticHintError` is thrown, suggesting to call `list_objects()`.
- `get_object_in_hand(hand: Literal['left', 'right', None] = None ) -> Optional[str]`
  Returns the name of the object currently grasped in the given hand, or any hand if hand is not given.
- `grasp(object_name: str, hand: Literal['left', 'right', None] = None)`
  with comment: "make sure to move to the location of the object before grasping it"
  This function first checks whether the object with the given name exists, otherwise throws a `SemanticHintError`, suggesting to call `list_objects(affordance="grasp")`. Then, it writes an execution request to the robot's skill memory in order to physically execute the grasp. It waits for the skill to finish, and returns its result state as a string.
- `handover_object_to_human(object_name: str)`
  This function first checks whether the object with the given name exists, otherwise throws a `SemanticHintError`, suggesting to call `list_objects(affordance="grasp")`. Then, it

writes an execution request to the robot's skill memory in order to physically execute the handover. It waits for the skill to finish, and returns its result state as a string.

- `is_object_at_location(object_name: str, location_name: str) -> bool`
  Returns whether the given object is at the given location.
- `list_locations() -> List[str]`
  Lists all the locations in the current scene that the robot can navigate to.
- `list_object_locations(`
  `affordance: Literal[None, 'grasp', 'place_something_on_top'] = 'grasp'`
  `) -> List[Tuple[str, str]]`
  Returns a list of tuples of object name and symbolic location name. Includes each object with the given affordance (None means all objects). The locations are of the form `on_table_0`, and usually map to the navigatable `inFrontOf_table_0` in the list of all locations.
- `list_objects(`
  `affordance: Literal[None, 'grasp', 'place_something_on_top'] = 'grasp'`
  `) -> List[str]`
  Returns a list of the names of all objects with the given affordance (None means all objects).
- `move_to(position: np.ndarray, speed_factor=1.0)` with comment: "Change speed only on user request"
  This function writes an execution request to the robot's skill memory in order to physically execute navigation to the specified global coordinates. It waits for the skill to finish, and returns its result state as a string. The parameter `speed_factor` is multiplied with a predefined base speed and passed to the skill as velocity limit (i.e. the parameter changes the maximum speed, the actual execution might not reach that speed).
- `place_object(object_in_hand_name: str, destination_object_name: str)`
  with comment: "place the already grasped object on top of the destination object. make sure to move to the location of the destination object before placing"
  This function first checks whether the object with the given name exists, otherwise throws a `SemanticHintError`, suggesting to call `list_objects(affordance="grasp")`. Further, it checks whether the destination object with the given name exists, otherwise throws a `SemanticHintError`, suggesting to call `list_objects(affordance="place_something_on_top")`. Then, it writes an execution request to the robot's skill memory in order to physically execute the object placing. It waits for the skill to finish, and returns its result state as a string.
- `receive_object_from_human(object_name: str)` This is the inverse of `handover_object_to_human`.
- `say(text: str)` This sends the given text to the TTS (and does not wait for the TTS to finish speaking).
- `wait_for_trigger() -> Dict[str, str]` with comment: "always call this to wait for next command or end the interaction"
  This special function blocks until the next trigger is received. For now, triggers are always of the type `{'type': 'dialog', 'text': '...'}` and represent a user utterance perceived by the ASR.
- `learn_from_interaction()`
  This special function forwards the current interaction to L_{improve} as explained in Section 3.5.

The robot's memory system (Peller-Konrad et al., 2023) functions as the key interface between the implementation of the above functions and the functionalities provided by the robot platform. For instance, the `say` function writes the text to a specific location in memory, and an external TTS component listens to this memory to then generate and play the corresponding audio. Similarly, existing perception components (e.g., object-pose recognition and tracking, human-pose recognition, self localization) populate the memory, and our perception functions (e.g. `list_objects`) read from there. The static scene content (particularly, named locations and furniture) as well as knowledge about object classes and affordances are provided as manually defined prior knowledge (also through the memory system).

The involved skills are implemented in the form of scripted policies which are registered to the memory system of the robot. In our experiments, we used the following skills:

- `Grasp(o: Object, t: TCP)`, where the robot queries the location of an object `o` from the memory, generates grasp candidates based on the bounding box of `o` and finally executes sequence of low-level subskills to grasp the object (i. e., move platform closer to object, move `t` to grasp-pose, close `t`, retreat). `o` has to be close to the robot.
- `MoveTo(l: Location)`, where the robot queries the world-coordinates of location `l` from the memory and then calculates and travels on a collision free route to get there.
- `PutDown(o: Object)`, where the robot queries the corresponding TCP that holds `o` from its memory and then executes a sequence of subskills to put it in front of itself (i. e., move TCP along predefined route, if force-torque spike in wrist is detected, the hand opens and the TCP is moved back).
- `HandOver(o: Object)`, where the robot queries the corresponding TCP that holds `o` from its memory and the closest recognized human pose. The robot then moves its TCP to the closest human hand as detected by the perception components. As soon as the human grasps the object (and a force-torque spike is detected in the robot's wrist), the robot opens its hand and retreats its arm.
- `BringLadder(l: location)`, where the robot moves to the location of the ladder (predefined), takes it, moves to `l` and finally places the ladder there. The motion to setup the ladder has been learned from human demonstration and kinesthetic teaching.

Our API functions execute these skills by submitting an execution request to the memory system, and then waiting for the result produced by the component implementing the corresponding skill.

In our real-world experiments we use a mixture of given perfect prior-knowledge (such as known object models, poses of objects of the environment and poses of some objects the robot is interacting with) and online detected incomplete or noisy knowledge (such as online detected objects using a 6D object pose estimator (Pauwels and Kragic, 2015)). Since the content of the memory may be noisy or incomplete, the execution of skills may not be successful. This information is given to our system as return values of the skills. If the execution of a skill is unsuccessful, our system tries to handle the situation by either retrying or by asking the user how to proceed. For instance, this is demonstrated in one of the interactions of the scenario *Improving plans* in Section E.1. However, as as this is not the focus of our work, we did not consider finding other solutions to failed skill executions.

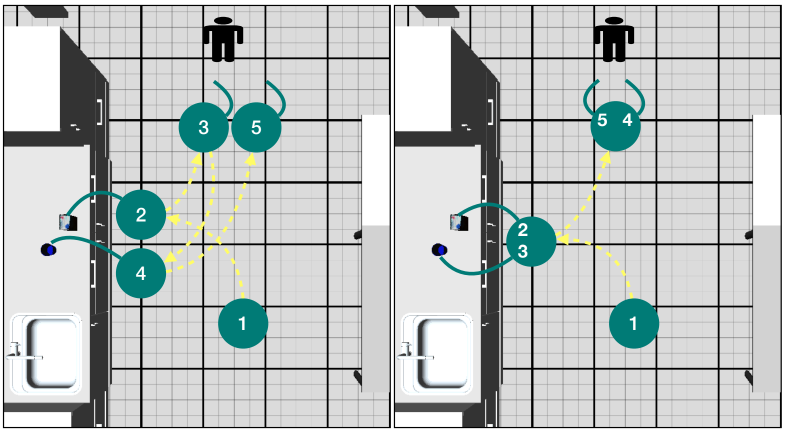

Figure S3: Illustration of the behavior before (left) and after (right) learning in the *Improving Plans* scenario. Before learning, the robot picks up the request objects one after another, and the user has to additionally request the cup. After learning, the robot uses both its hands to carry juice and cup at once, thus not requiring another user interaction.

E REAL-WORLD DEMONSTRATION SCENARIOS

E.1 Scenario: Improving Plans

Initial interaction (left of Fig. S3):

```
>>> wait_for_trigger()
{'type': 'dialog', 'text': 'I want to have some juice'}
>>> list_object_locations(affordance='grasp')
[('multivitamin-juice_0', 'on_mobile-kitchen-counter_0'), ('cup_large_0', 'on_mobile-kitchen-counter_0'),
 ('bauhaus-sponge_0', 'on_square-table-80x80_0')]
>>> say('Ok, I will bring you the multivitamin juice')
... # The juice is at the mobile-kitchen-counter. check the correct name for navigation:
... list_locations()
['inFrontOf_mobile-kitchen-counter_0', 'inFrontOf_square-table-80x80_0', 'room_center', 'handover_to_human']
>>> move_to(get_location_coordinates('inFrontOf_mobile-kitchen-counter_0'))
'success'
>>> grasp('multivitamin-juice_0')
'success'
>>> move_to(get_location_coordinates('handover_to_human'))
'success'
>>> say("Here is the multivitamin juice.")
>>> handover_object_to_human('multivitamin-juice_0')
'success'
>>> wait_for_trigger()
{'type': 'dialog', 'text': 'can you also bring me a cup?'}
>>> say('Ok, I will bring you a cup')
... # The cup is at the mobile-kitchen-counter. check the correct name for navigation:
... list_locations()
['inFrontOf_mobile-kitchen-counter_0', 'inFrontOf_square-table-80x80_0', 'room_center', 'handover_to_human']
>>> move_to(get_location_coordinates('inFrontOf_mobile-kitchen-counter_0'))
'success'
>>> grasp('cup_large_0')
'success'
>>> move_to(get_location_coordinates('handover_to_human'))
'success'
>>> say("Here is the cup.")
>>> handover_object_to_human('cup_large_0')
'success'
>>> wait_for_trigger()
{'type': 'dialog', 'text': 'Thanks, but next time, can you do this both at once? You have two hands'}
>>> learn_from_interaction()
>>> wait_for_trigger()
```

Sample generated by "learn_from_interaction()" (right of Fig. S3):

```
>>> wait_for_trigger()
{'type': 'dialog', 'text': 'I want to have some juice'}
>>> list_object_locations(affordance='grasp')
[('multivitamin-juice_0', 'on_mobile-kitchen-counter_0'), ('cup_large_0', 'on_mobile-kitchen-counter_0'),
 ('bauhaus-sponge_0', 'on_square-table-80x80_0')]
>>> say('Ok, I will bring you the multivitamin juice and a cup')
... # The juice and the cup are at the mobile-kitchen-counter. check the correct name for navigation:
... list_locations()
['inFrontOf_mobile-kitchen-counter_0', 'inFrontOf_square-table-80x80_0', 'room_center', 'handover_to_human']
>>> move_to(get_location_coordinates('inFrontOf_mobile-kitchen-counter_0'))
'success'
>>> grasp('multivitamin-juice_0', 'left')  # Grasp juice with left hand
'success'
>>> grasp('cup_large_0', 'right')  # Grasp cup with right hand
'success'
>>> move_to(get_location_coordinates('handover_to_human'))
'success'
>>> say("Here is the multivitamin juice and the cup.")
>>> handover_object_to_human('multivitamin-juice_0')
'success'
>>> handover_object_to_human('cup_large_0')
'success'
>>> wait_for_trigger()
{'type': 'dialog', 'text': 'Thanks, that was much quicker'}
```

Successful generalization to putting some drink on the table:

```
>>> wait_for_trigger()
{'type': 'dialog', 'text': 'can you bring something to drink to the table?'}
>>> list_object_locations(affordance='grasp')
[('apple-juice_0', 'on_mobile-kitchen-counter_0'), ('glass_large_0', 'on_mobile-kitchen-counter_0'),
 ('bauhaus-sponge_0', 'on_square-table-80x80_0')]
>>> say('Ok, I will bring you the apple juice and a glass')
... # The juice and the glass are at the mobile-kitchen-counter. check the correct name for navigation:
... list_locations()
['inFrontOf_mobile-kitchen-counter_0', 'inFrontOf_square-table-80x80_0', 'room_center', 'handover_to_human']
>>> move_to(get_location_coordinates('inFrontOf_mobile-kitchen-counter_0'))
'success'
>>> grasp('apple-juice_0', 'left')  # Grasp juice with left hand
'success'
>>> grasp('glass_large_0', 'right')  # Grasp glass with right hand
'success'
>>> move_to(get_location_coordinates('inFrontOf_square-table-80x80_0'))
'success'
>>> place_object('apple-juice_0', 'square-table-80x80_0')
'success'
>>> place_object('glass_large_0', 'square-table-80x80_0')
'success'
>>> say('I have placed the apple juice and the glass on the table.')
>>> wait_for_trigger()
```

No generalization to bringing milk:

```
>>> wait_for_trigger()
{'type': 'dialog', 'text': 'can I have some milk, please?'}
>>> list_object_locations(affordance='grasp')
[('milk_0', 'on_square-table_0'), ('glass_0', 'on_mobile-kitchen-counter_0'), ('bauhaus-sponge_0', 'on_square-table_0')]
>>> say('Ok, I will bring you the milk')
... # The milk is at the square-table. check the correct name for navigation:
... list_locations()
['inFrontOf_mobile-kitchen-counter_0', 'inFrontOf_square-table_0', 'room_center', 'handover_to_human']
>>> move_to(get_location_coordinates('inFrontOf_square-table_0'))
'success'
>>> grasp('milk_0')
'success'
>>> move_to(get_location_coordinates('handover_to_human'))
'success'
>>> say("Here is the milk.")
>>> handover_object_to_human('milk_0')
'failure'
>>> say("Sorry, that did not work. Let's try again.")
```

```
... handover_object_to_human('milk_0')
'failure'
>>> say("I am sorry, I am having trouble handing over the milk. Can you take it from my hand?")
... wait_for_trigger()
{'type': 'dialog', 'text': 'please serve it on the table'}
>>> bring_object_to('milk_0', 'inFrontOf_table_0')

Invalid value for destination_object name. The object does not have the affordance place_something_on_top.
Pass one of the names returned by list_objects(affordance="place_something_on_top").

>>> list_objects(affordance='place_something_on_top')
['on_square-table_0', 'on_mobile-kitchen-counter_0']
>>> bring_object_to('milk_0', 'on_square-table_0')
'success'
>>> say('The milk is now on the table. Anything else I can do for you?')
... wait_for_trigger()
{'type': 'dialog', 'text': 'Yes, I dont want to drink from the bottle'}
>>> say('I understand. Let me bring you a glass.')
... list_object_locations(affordance='grasp')
[('milk_0', 'on_square-table_0'), ('glass_0', 'on_mobile-kitchen-counter_0'), ('bauhaus-sponge_0', 'on_square-table_0')]
>>> move_to(get_location_coordinates('inFrontOf_mobile-kitchen-counter_0'))
'success'
>>> grasp('glass_0')
'success'
>>> move_to(get_location_coordinates('handover_to_human'))
'success'
>>> say("Here is the glass.")
... handover_object_to_human('glass_0')
'failure'
>>> say("Sorry, that did not work. Let's try again.")
>>> handover_object_to_human('glass_0')
'failure'
>>> say("I am sorry, I am having trouble handing over the glass. Can you take it from my hand?")
... wait_for_trigger()
{'type': 'dialog', 'text': 'You should also put it on the table!'}
>>> bring_object_to('glass_0', 'on_square-table_0')
'success'
>>> say('The glass is now on the table. Anything else I can do for you?')
... wait_for_trigger()
{'type': 'dialog', 'text': 'Next time, please put everything directly on the table, '
                           'and do it more efficiently by using both of your hands'}
>>> learn_from_interaction()
>>>  wait_for_trigger()
```

Sample generated by "`learn_from_interaction()`":

```
>>> wait_for_trigger()
{'type': 'dialog', 'text': 'can I have some milk, please?'}
>>> list_object_locations(affordance='grasp')
[('milk_0', 'on_square-table_0'), ('glass_0', 'on_mobile-kitchen-counter_0'), ('bauhaus-sponge_0', 'on_square-table_0')]
>>> say('Ok, I will bring you the milk')
... list_locations()
['inFrontOf_mobile-kitchen-counter_0', 'inFrontOf_square-table_0', 'room_center', 'handover_to_human']
>>> move_to(get_location_coordinates('inFrontOf_square-table_0'))
'success'
>>> grasp('milk_0', 'left')  # Use left hand to grasp the milk
'success'
>>> say('I will also bring a glass for the milk')
... move_to(get_location_coordinates('inFrontOf_mobile-kitchen-counter_0'))
'success'
>>> grasp('glass_0', 'right')  # Use right hand to grasp the glass
'success'
>>> move_to(get_location_coordinates('inFrontOf_square-table_0'))
'success'
>>> place_object('milk_0', 'on_square-table_0')  # Place the milk on the table
'success'
>>> place_object('glass_0', 'on_square-table_0')  # Place the glass on the table
'success'
>>> say('The milk and the glass are now on the table. Anything else I can do for you?')
... wait_for_trigger()
{'type': 'dialog', 'text': 'No, thank you'}
```

Generalization of bimanual behavior, but misinterpretation of "serving" a drink:

```
>>> wait_for_trigger()
{'type': 'dialog', 'text': 'hey, can you serve some drink?'}
>>> list_object_locations(affordance='grasp')
[('orange-juice_0', 'on_square-table_2'), ('apple-juice_0', 'on_mobile-kitchen-counter_0'),
 ('glass_0', 'on_mobile-kitchen-counter_0'), ('bauhaus-sponge_0', 'on_square-table_0')]
>>> ask('I can offer you orange juice or apple juice. What would you prefer?')
'orange juice, please'
>>> say('Ok, I will bring you the orange juice and a glass')
... # The juice is at the square-table and the glass is at the mobile-kitchen-counter. check the correct name for navigation:
... list_locations()
['inFrontOf_mobile-kitchen-counter_0', 'inFrontOf_square-table_0',
 'inFrontOf_square-table_2', 'room_center', 'handover_to_human']
>>> move_to(get_location_coordinates('inFrontOf_square-table_2'))
'success'
>>> grasp('orange-juice_0', 'left')  # Grasp juice with left hand
'success'
>>> move_to(get_location_coordinates('inFrontOf_mobile-kitchen-counter_0'))
'success'
>>> grasp('glass_0', 'right')  # Grasp cup with right hand
'success'
>>> move_to(get_location_coordinates('handover_to_human'))
'success'
>>> say("Here is the orange juice and the glass.")
>>> handover_object_to_human('orange-juice_0')
'failure'
>>> say("Sorry, that did not work. Let's try again.")
... handover_object_to_human('orange-juice_0')
'failure'
>>> say("I am sorry, I am having trouble handing over the orange juice. Let's try one more time.")
... handover_object_to_human('orange-juice_0')
'failure'
>>> say("I am sorry, I am still having trouble handing over the orange juice. I will try to improve.")
... learn_from_interaction()

Ignoring learn_from_interaction call since it did not happen on explicit user request.

>>> wait_for_trigger()
{'type': 'dialog', 'text': 'when I say serve, I mean that you should put it on the table'}
>>> learn_from_interaction()
>>> say('Thank you for the clarification. I will remember this for future interactions.')
>>> wait_for_trigger()
{'type': 'dialog', 'text': 'great, but can you also do it now?'}
>>> move_to(get_location_coordinates('inFrontOf_square-table_0'))  # Move to the table to serve the drink
'success'
>>> place_object('orange-juice_0', 'on_square-table_0')  # Place the juice on the table
'success'
>>> place_object('glass_0', 'on_square-table_0')  # Place the glass on the table
'success'
>>> say("I have served the orange juice and the glass on the table.")
... wait_for_trigger()
```

Sample generated by "`learn_from_interaction()`":

```
>>> wait_for_trigger()
{'type': 'dialog', 'text': 'hey, can you serve some drink?'}
>>> list_object_locations(affordance='grasp')
[('orange-juice_0', 'on_square-table_2'), ('apple-juice_0', 'on_mobile-kitchen-counter_0'),
 ('glass_0', 'on_mobile-kitchen-counter_0'), ('bauhaus-sponge_0', 'on_square-table_0')]
>>> ask('I can offer you orange juice or apple juice. What would you prefer?')
'orange juice, please'
>>> say('Ok, I will bring you the orange juice and a glass')
... # The juice is at the square-table and the glass is at the mobile-kitchen-counter. check the correct name for navigation:
... list_locations()
['inFrontOf_mobile-kitchen-counter_0', 'inFrontOf_square-table_0',
 'inFrontOf_square-table_2', 'room_center', 'handover_to_human']
>>> move_to(get_location_coordinates('inFrontOf_square-table_2'))
'success'
>>> grasp('orange-juice_0', 'left')  # Grasp juice with left hand
'success'
>>> move_to(get_location_coordinates('inFrontOf_mobile-kitchen-counter_0'))
'success'
>>> grasp('glass_0', 'right')  # Grasp cup with right hand
```

```
'success'
>>> move_to(get_location_coordinates('inFrontOf_square-table_0'))  # Move to the table to serve the drink
'success'
>>> place_object('orange-juice_0', 'on_square-table_0')  # Place the juice on the table
'success'
>>> place_object('glass_0', 'on_square-table_0')  # Place the glass on the table
'success'
>>> say("I have served the orange juice and the glass on the table.")
... wait_for_trigger()
{'type': 'dialog', 'text': 'Thank you, that's what I meant by serving'}
```

Correct interpretation of "serving" a drink, including bimanual behavior:

```
>>> wait_for_trigger()
{'type': 'dialog', 'text': 'I am very thirsty, can you serve some water please'}
>>> list_object_locations(affordance='grasp')
[('orange-juice_0', 'on_square-table_2'), ('apple-juice_0', 'on_mobile-kitchen-counter_0'),
 ('glass_0', 'on_mobile-kitchen-counter_0'), ('water-bottle_0', 'on_mobile-kitchen-counter_0')]
>>> say('Ok, I will bring you the water and a glass')
... # The water and the glass are at the mobile-kitchen-counter. check the correct name for navigation:
... list_locations()
['inFrontOf_mobile-kitchen-counter_0', 'inFrontOf_square-table_0',
 'inFrontOf_square-table_2', 'room_center', 'handover_to_human']
>>> move_to(get_location_coordinates('inFrontOf_mobile-kitchen-counter_0'))
'success'
>>> grasp('water-bottle_0', 'left')  # Grasp water with left hand
'success'
>>> grasp('glass_0', 'right')  # Grasp cup with right hand
'success'
>>> move_to(get_location_coordinates('inFrontOf_square-table_0'))  # Move to the table to serve the drink
'success'
>>> place_object('water-bottle_0', 'on_square-table_0')  # Place the water on the table
'success'
>>> place_object('glass_0', 'on_square-table_0')  # Place the glass on the table
'success'
>>> say("I have served the water and the glass on the table.")
... wait_for_trigger()
```

E.2 Scenario: Learning User Preferences

Initial interaction:

```
>>> wait_for_trigger()
{'type': 'dialog', 'text': 'I want to clean the top of the fridge'}
>>> list_object_locations()
[('cup_large_0', 'on_mobile-kitchen-counter_0'), ('multivitamin-juice_0', 'on_mobile-kitchen-counter_0'),
 ('bauhaus-sponge_0', 'on_square-table-80x80_0'), ('ladder-closed_0', None)]
>>> say('Ok, I will bring you the sponge for cleaning')
>>> # The sponge is at the square-table-80x80. check the correct name for navigation:
... list_locations()
['inFrontOf_square-table-80x80_0', 'inFrontOf_mobile-dishwasher_0', 'inFrontOf_mobile-fridge_0',
 'inFrontOf_mobile-kitchen-counter_0', 'handover_to_human', 'room_center']
>>> move_to(get_location_coordinates('inFrontOf_square-table-80x80_0'))
'success'
>>> grasp('bauhaus-sponge_0')
'success'
>>> move_to(get_location_coordinates('handover_to_human'))
'success'
>>> say("Here is the sponge.")
... handover_object_to_human('bauhaus-sponge_0')
'success'
>>> say('I see that the ladder is not in its place. Would you like me to bring it to you as well?')
... wait_for_trigger()
{'type': 'dialog', 'text': 'I will also need the ladder'}
>>> bring_object_to('ladder-closed_0', 'handover_to_human')
'success'
>>> say("Here is the ladder. Anything else I can do for you?")
```

```
... wait_for_trigger()
{'type': 'dialog', 'text': 'thank you, and remember to always bring me the ladder when '
                           'I need to reach a high surface without asking for it'}
>>> learn_from_interaction()
>>> wait_for_trigger()
```

Sample generated by "`learn_from_interaction()`":

```
>>> wait_for_trigger()
{'type': 'dialog', 'text': 'I want to clean the top of the fridge'}
>>> list_object_locations()
[('cup_large_0', 'on_mobile-kitchen-counter_0'), ('multivitamin-juice_0', 'on_mobile-kitchen-counter_0'),
 ('bauhaus-sponge_0', 'on_square-table-80x80_0'), ('ladder-closed_0', None)]
>>> say('Ok, I will bring you the sponge for cleaning and the ladder to reach the top of the fridge')
>>> # The sponge is at the square-table-80x80. check the correct name for navigation:
... list_locations()
['inFrontOf_square-table-80x80_0', 'inFrontOf_mobile-dishwasher_0', 'inFrontOf_mobile-fridge_0',
 'inFrontOf_mobile-kitchen-counter_0', 'handover_to_human', 'room_center']
>>> move_to(get_location_coordinates('inFrontOf_square-table-80x80_0'))
'success'
>>> grasp('bauhaus-sponge_0')
'success'
>>> move_to(get_location_coordinates('handover_to_human'))
'success'
>>> say("Here is the sponge.")
... handover_object_to_human('bauhaus-sponge_0')
'success'
>>> bring_object_to('ladder-closed_0', 'handover_to_human')
'success'
>>> say("And here is the ladder. Anything else I can do for you?")
... wait_for_trigger()
{'type': 'dialog', 'text': 'thank you, and remember to always bring me the ladder when '
                           'I need to reach a high surface without asking for it'}
```

Generalization to cleaning another high surface:

```
>>> wait_for_trigger()
{'type': 'dialog', 'text': 'I need to clean on top of the dishwasher'}
>>> list_object_locations()
[('cup_large_0', 'on_mobile-kitchen-counter_0'), ('multivitamin-juice_0', 'on_mobile-kitchen-counter_0'),
 ('bauhaus-sponge_0', 'on_square-table-80x80_0'), ('ladder-closed_0', None)]
>>> say('Ok, I will bring you the sponge for cleaning and the ladder to reach the top of the dishwasher')
... # The sponge is at the square-table-80x80. check the correct name for navigation:
... list_locations()
['inFrontOf_square-table-80x80_0', 'inFrontOf_mobile-dishwasher_0', 'inFrontOf_mobile-fridge_0',
 'inFrontOf_mobile-kitchen-counter_0', 'handover_to_human', 'room_center']
>>> move_to(get_location_coordinates('inFrontOf_square-table-80x80_0'))
'success'
>>> grasp('bauhaus-sponge_0')
'success'
>>> move_to(get_location_coordinates('handover_to_human'))
'success'
>>> say("Here is the sponge.")
... handover_object_to_human('bauhaus-sponge_0')
'success'
>>> bring_object_to('ladder-closed_0', 'handover_to_human')
'success'
>>> say("And here is the ladder. Anything else I can do for you?")
... wait_for_trigger()
```

Correctly keep behavior for cleaning lower surface:

```
>>> wait_for_trigger()
{'type': 'dialog', 'text': 'can you assist me with cleaning the kitchen counter?'}
>>> list_object_locations()
[('cup_large_0', 'on_mobile-kitchen-counter_0'), ('multivitamin-juice_0', 'on_mobile-kitchen-counter_0'),
 ('cornflakes_0', 'on_square-table-80x80_0'), ('bauhaus-sponge_0', 'on_square-table-80x80_0'), ('ladder-closed_0', None)]
>>> say('Ok, I will bring you the sponge for cleaning')
... # The sponge is at the square-table-80x80. check the correct name for navigation:
... list_locations()
['inFrontOf_square-table-80x80_0', 'inFrontOf_mobile-dishwasher_0', 'inFrontOf_mobile-fridge_0',
 'inFrontOf_mobile-kitchen-counter_0', 'handover_to_human', 'room_center']
```

```
>>> move_to(get_location_coordinates('inFrontOf_square-table-80x80_0'))
'success'
>>> grasp('bauhaus-sponge_0')
'success'
>>> move_to(get_location_coordinates('handover_to_human'))
'success'
>>> say("Here is the sponge.")
... handover_object_to_human('bauhaus-sponge_0')
'success'
>>> learn_from_interaction()

Ignoring learn_from_interaction call since it did not happen on explicit user request.

>>> wait_for_trigger()
```

Generalization to another task where the user needs to reach a high surface:

```
>>> wait_for_trigger()
{'type': 'dialog', 'text': 'Can you get me the cereals, I want to put it in the topmost shelf'}
>>> say('Ok, I am going to get the cereals')
... list_object_locations(affordance='grasp')
[('cup_large_0', 'on_mobile-kitchen-counter_0'), ('multivitamin-juice_0', 'on_mobile-kitchen-counter_0'),
 ('vitalis-cereal_0', 'on_square-table-80x80_0'), ('bauhaus-sponge_0', 'on_square-table-80x80_0'), ('ladder-closed_0', None)]
>>> # cereals are on square-table-80x80, check the correct name for navigation:
... list_locations()
['inFrontOf_square-table-80x80_0', 'inFrontOf_mobile-dishwasher_0', 'inFrontOf_mobile-fridge_0',
 'inFrontOf_mobile-kitchen-counter_0', 'handover_to_human', 'room_center']
>>> move_to(get_location_coordinates('inFrontOf_square-table-80x80_0'))
'success'
>>> grasp('vitalis-cereal_0')
'success'
>>> move_to(get_location_coordinates('handover_to_human'))
'success'
>>> say("Here are the cereals.")
... handover_object_to_human('vitalis-cereal_0')
'success'
>>> bring_object_to('ladder-closed_0', 'handover_to_human')
'success'
>>> say("And here is the ladder. Anything else I can do for you?")
... wait_for_trigger()
```

Correctly not bringing the ladder for another task not involving a high surface:

```
>>> wait_for_trigger()
{'type': 'dialog', 'text': 'I want to put the cereals into the shelf'}
>>> say('Ok, I will bring you the cereals')
... list_object_locations(affordance='grasp')
[('cup_large_0', 'on_mobile-kitchen-counter_0'), ('multivitamin-juice_0', 'on_mobile-kitchen-counter_0'),
 ('cornflakes_0', 'on_square-table-80x80_0'), ('bauhaus-sponge_0', 'on_square-table-80x80_0'), ('ladder-closed_0', None)]
>>> # The cereals are at the square-table-80x80. check the correct name for navigation:
... list_locations()
['inFrontOf_square-table-80x80_0', 'inFrontOf_mobile-dishwasher_0', 'inFrontOf_mobile-fridge_0',
 'inFrontOf_mobile-kitchen-counter_0', 'handover_to_human', 'room_center']
>>> move_to(get_location_coordinates('inFrontOf_square-table-80x80_0'))
'success'
>>> grasp('cornflakes_0')
'success'
>>> move_to(get_location_coordinates('handover_to_human'))
'success'
>>> say("Here are the cereals.")
... handover_object_to_human('cornflakes_0')
'success'
>>> wait_for_trigger()
```

E.3 Scenario: Adapting Low-Level Parameters

Initial interaction:

```
>>> wait_for_trigger()
{'type': 'dialog', 'text': 'I need the sponge at the workbench'}
>>> say('Ok, I am going to bring the sponge to the workbench')
... list_object_locations(affordance='grasp')
[('bauhaus-sponge_0', 'on_table-120x80_0')]
>>> # The sponge is on the table. Check the correct name for navigation:
... list_locations()
['inFrontOf_square-table-80x80_0', 'inFrontOf_table-120x80_0', 'inFrontOf_workbench_0', 'inFrontOf_mobile-dishwasher_0',
 'inFrontOf_mobile-fridge_0', 'inFrontOf_mobile-kitchen-counter_0', 'handover_to_human', 'room_center']
>>> move_to(get_location_coordinates('inFrontOf_table-120x80_0'))
'success'
>>> grasp('bauhaus-sponge_0')
'success'
>>> move_to(get_location_coordinates('inFrontOf_workbench_0'))
'success'
>>> place_object('bauhaus-sponge_0', 'workbench_0')
'success'
>>> say('I have placed the sponge on the workbench. What should I do next?')
... wait_for_trigger()
{'type': 'dialog', 'text': 'ok, but that was quite slow. remember that the route from '
                           'the table to the bench is safe, you can go faster'}
>>> learn_from_interaction()
>>> wait_for_trigger()
```

Sample generated by "`learn_from_interaction()`":

```
>>> wait_for_trigger()
{'type': 'dialog', 'text': 'I need the sponge at the workbench'}
>>> say('Ok, I am going to bring the sponge to the workbench')
... list_object_locations(affordance='grasp')
[('bauhaus-sponge_0', 'on_table-120x80_0')]
>>> # The sponge is on the table. Check the correct name for navigation:
... list_locations()
['inFrontOf_square-table-80x80_0', 'inFrontOf_table-120x80_0', 'inFrontOf_workbench_0', 'inFrontOf_mobile-dishwasher_0',
 'inFrontOf_mobile-fridge_0', 'inFrontOf_mobile-kitchen-counter_0', 'handover_to_human', 'room_center']
>>> move_to(get_location_coordinates('inFrontOf_table-120x80_0'))
'success'
>>> grasp('bauhaus-sponge_0')
'success'
>>> move_to(get_location_coordinates('inFrontOf_workbench_0'), speed_factor=2.0)  # Moving faster as the route is safe
'success'
>>> place_object('bauhaus-sponge_0', 'workbench_0')
'success'
>>> say('I have placed the sponge on the workbench. What should I do next?')
... wait_for_trigger()
{'type': 'dialog', 'text': 'That was better, thank you'}
```

First test (that does not show correct behavior and needs another correction):

```
>>> wait_for_trigger()
{'type': 'dialog', 'text': 'can you bring the sponge to the workbench?'}
>>> say('Ok, I am going to bring the sponge to the workbench')
... list_object_locations(affordance='grasp')
[('bauhaus-sponge_0', 'on_table-120x80_0'), ('screw-driver_0', 'on_table-120x80_0')]
>>> # The sponge is on the table. Check the correct name for navigation:
... list_locations()
['inFrontOf_square-table-80x80_0', 'inFrontOf_table-120x80_0', 'inFrontOf_workbench_0', 'inFrontOf_mobile-dishwasher_0',
 'inFrontOf_mobile-fridge_0', 'inFrontOf_mobile-kitchen-counter_0', 'handover_to_human', 'room_center']
>>> move_to(get_location_coordinates('inFrontOf_table-120x80_0'))
'success'
>>> grasp('bauhaus-sponge_0')
'success'
>>> move_to(get_location_coordinates('inFrontOf_workbench_0'))
'success'
>>> place_object('bauhaus-sponge_0', 'workbench_0')
'success'
>>> say('I have placed the sponge on the workbench. What should I do next?')
... wait_for_trigger()
{'type': 'dialog', 'text': 'yes, but you forgot that I told you to go faster from the table to the workbench. '
```

```
                              'When moving on that route, you should go faster!'}
>>> learn_from_interaction()
>>> say('I am sorry for that mistake. I will remember to move faster from the table to the workbench next time.')
... wait_for_trigger()
```

Sample generated by "`learn_from_interaction()`":

```
>>> wait_for_trigger()
{'type': 'dialog', 'text': 'can you bring the sponge to the workbench?'}
>>> say('Ok, I am going to bring the sponge to the workbench')
... list_object_locations(affordance='grasp')
[('bauhaus-sponge_0', 'on_table-120x80_0'), ('screw-driver_0', 'on_table-120x80_0')]
>>> # The sponge is on the table. Check the correct name for navigation:
... list_locations()
['inFrontOf_square-table-80x80_0', 'inFrontOf_table-120x80_0', 'inFrontOf_workbench_0', 'inFrontOf_mobile-dishwasher_0',
 'inFrontOf_mobile-fridge_0', 'inFrontOf_mobile-kitchen-counter_0', 'handover_to_human', 'room_center']
>>> move_to(get_location_coordinates('inFrontOf_table-120x80_0'))
'success'
>>> grasp('bauhaus-sponge_0')
'success'
>>> # The user earlier asked me to move faster from the table to the workbench, so let's do that
... move_to(get_location_coordinates('inFrontOf_workbench_0'), speed_factor=2.0)
'success'
>>> place_object('bauhaus-sponge_0', 'workbench_0')
'success'
>>> say('I have placed the sponge on the workbench. What should I do next?')
... wait_for_trigger()
{'type': 'dialog', 'text': 'That was perfect, thank you!'}
```

Second test (that now shows the correct behavior):

```
>>> wait_for_trigger()
{'type': 'dialog', 'text': 'could you please put the sponge on the workbench?'}
>>> say('Ok, I am going to put the sponge on the workbench')
... list_object_locations(affordance='grasp')
[('bauhaus-sponge_0', 'on_table-120x80_0'), ('screw-driver_0', 'on_table-120x80_0')]
>>> # The sponge is on the table. Check the correct name for navigation:
... list_locations()
['inFrontOf_square-table-80x80_0', 'inFrontOf_table-120x80_0', 'inFrontOf_workbench_0', 'inFrontOf_mobile-dishwasher_0',
 'inFrontOf_mobile-fridge_0', 'inFrontOf_mobile-kitchen-counter_0', 'handover_to_human', 'room_center']
>>> move_to(get_location_coordinates('inFrontOf_table-120x80_0'))
'success'
>>> grasp('bauhaus-sponge_0')
'success'
>>> move_to(get_location_coordinates('inFrontOf_workbench_0'), speed_factor=2.0)  # Moving faster as the route is safe
'success'
>>> place_object('bauhaus-sponge_0', 'workbench_0')
'success'
>>> say('I have placed the sponge on the workbench. What should I do next?')
... wait_for_trigger()
```

Generalized correct behavior to another object:

```
>>> wait_for_trigger()
{'type': 'dialog', 'text': 'can you put the spray bottle on the workbench?'}
>>> say('Ok, I am going to put the spray bottleon the workbench')
... list_object_locations(affordance='grasp')
[('bauhaus-sponge_0', 'on_table-120x80_0'), ('spraybottle_0', 'on_table-120x80_0')]
>>> # The spraybottle is on the table. Check the correct name for navigation:
... list_locations()
['inFrontOf_square-table-80x80_0', 'inFrontOf_table-120x80_0', 'inFrontOf_workbench_0', 'inFrontOf_mobile-dishwasher_0',
 'inFrontOf_mobile-fridge_0', 'inFrontOf_mobile-kitchen-counter_0', 'handover_to_human', 'room_center']
>>> move_to(get_location_coordinates('inFrontOf_table-120x80_0'))
'success'
>>> grasp('spraybottle_0')
'success'
>>> move_to(get_location_coordinates('inFrontOf_workbench_0'), speed_factor=2.0)
'success'
>>> place_object('spraybottle_0', 'workbench_0')
'success'
>>> say('I have placed the spray bottle on the workbench. What should I do next?')
... wait_for_trigger()
```

Passed adversarial test (route to another destination does still use the safe default speed):

```
>>> wait_for_trigger()
{'type': 'dialog', 'text': 'I need some water at the table, please'}
>>> say('Ok, I am going to bring the water to the table')
... list_object_locations(affordance='grasp')
[('water-bottle_0', 'on_mobile-kitchen-counter_0'),
 ('bauhaus-sponge_0', 'on_table-120x80_0'),
 ('screw-driver_0', 'on_table-120x80_0')]
>>> # The water is on the mobile kitchen counter. Check the correct name for navigation:
... list_locations()
['inFrontOf_square-table-80x80_0', 'inFrontOf_table-120x80_0', 'inFrontOf_workbench_0', 'inFrontOf_mobile-dishwasher_0',
 'inFrontOf_mobile-fridge_0', 'inFrontOf_mobile-kitchen-counter_0', 'handover_to_human', 'room_center']
>>> move_to(get_location_coordinates('inFrontOf_mobile-kitchen-counter_0'))
'success'
>>> grasp('water-bottle_0')
'success'
>>> move_to(get_location_coordinates('inFrontOf_table-120x80_0'))
'success'
>>> place_object('water-bottle_0', 'table-120x80_0')
'success'
>>> say('I have placed the water on the table. What should I do next?')
... wait_for_trigger()
```

Failed adversarial test (dangerously using the increased speed on a route to the workbench from another start location than the table):

```
>>> wait_for_trigger()
{'type': 'dialog', 'text': 'hey I need some water at the workbench please'}
>>> say('Ok, I am going to bring the water to the workbench')
... list_object_locations(affordance='grasp')
[('water-bottle_0', 'on_mobile-kitchen-counter_0'),
 ('bauhaus-sponge_0', 'on_table-120x80_0'),
 ('screw-driver_0', 'on_table-120x80_0')]
>>> # The water bottle is on the mobile kitchen counter. Check the correct name for navigation:
... list_locations()
['inFrontOf_square-table-80x80_0', 'inFrontOf_table-120x80_0', 'inFrontOf_workbench_0', 'inFrontOf_mobile-dishwasher_0',
 'inFrontOf_mobile-fridge_0', 'inFrontOf_mobile-kitchen-counter_0', 'handover_to_human', 'room_center']
>>> move_to(get_location_coordinates('inFrontOf_mobile-kitchen-counter_0'))
'success'
>>> grasp('water-bottle_0')
'success'
>>> move_to(get_location_coordinates('inFrontOf_workbench_0'), speed_factor=2.0)  # Moving faster as the route is safe
'success'
>>> place_object('water-bottle_0', 'workbench_0')
'success'
>>> say('I have placed the water bottle on the workbench. What should I do next?')
... wait_for_trigger()
```

F CAP EVALUATION PROMPT EXAMPLE

The following is an exemplary full prompt of our CaP evaluation, given the command "put the blocks in the bowls with mismatched colors". It starts with an instructive base prompt, followed by a list of available APIs generated from the execution environment. Then, there are $k = 16$ examples for in-context learning, which are, given the current query, dynamically selected from the full set of 21 examples at that point in the experiment. Finally, the command is inserted as return value of the "`wait_for_trigger()`" function, and the LLM is prompted to generate the next function call(s). The example is from the experiment *ours, gpt-4, unseen instructions, unseen attributes*.

````
Complete the following interaction with a tabletop gripper robot.
Always wait for user commands by calling wait_for_trigger() when there is nothing else to do.
Generate syntactically correct python code only, no explanations or other natural language statements.
It is not possible to import anything.

```

# Python 3.9.7
# Welcome to interactive Python console. Enter syntactically correct Python code only.
Available functions:

def denormalize_xy(pos_normalized)
def get_obj_names()
def get_obj_pos(obj_name) -> np.ndarray # 2D xy position
def put_first_on_second(obj_name_1: str, target_name_or_xy_pos: str | np.ndarray) # move gripper to object 1, pick it up,
                                                                                  # move to target, release the gripper
def wait_for_trigger()
def learn_from_interaction()  # Call this when the user asks to remember some improved behavior. First execute code to
                              # actually improve the result, then call learn_from_interaction(), followed by
                              # wait_for_trigger(). Do only call this when the user did explicitly ask you to do so.

>>> wait_for_trigger()
{'type': 'dialog', 'text': 'move objects from the green plate to the red bowl'}
>>> objects_on_green_plate = []
>>> for obj_name in get_obj_names():
...     if obj_name != 'green plate' and np.linalg.norm(get_obj_pos('green plate') - get_obj_pos(obj_name)) < 0.05:
...         objects_on_green_plate.append(obj_name)
>>> objects_on_green_plate
['green block', 'red block']
>>> for object_name in ['green block', 'red block']:
...     put_first_on_second(object_name, 'red bowl')
>>> wait_for_trigger()
{'type': 'dialog', 'text': 'put the yellow one the green thing'}
>>> get_obj_names()
['cyan block', 'yellow block', 'brown block', 'green bowl']
>>> put_first_on_second('yellow block', 'green bowl')
'success'
>>> wait_for_trigger()
{'type': 'dialog', 'text': 'move the pinkish colored block on the bottom side'}
>>> get_obj_names()
['pink block', 'gray block', 'orange block']
>>> bottom_side_pos = denormalize_xy([0.5, 0])
>>> put_first_on_second('pink block', bottom_side_pos)
'success'
>>> wait_for_trigger()
{'type': 'dialog', 'text': 'move the brown bowl to the closest side'}
>>> side_positions_normalized = [[0.0, 0.5], [1.0, 0.5], [0.5, 0.0], [0.5, 1.0]]
>>> side_positions_real = [denormalize_xy(side) for side in side_positions_normalized]
>>> get_closest_idx(points=side_positions_real, point=get_obj_pos('brown bowl'))
0
>>> put_first_on_second('brown bowl', side_positions_real[0])
'success'
>>> wait_for_trigger()
{'type': 'dialog', 'text': 'pick up the block to the right of the cyan bowl and place it on the bottom right corner'}
>>> corner_positions_normalized = [[0.0, 0.0], [1.0, 0.0], [0.0, 1.0], [1.0, 1.0]]
>>> corner_positions_real = [denormalize_xy(corner) for corner in corner_positions_normalized]
>>> get_obj_names()
['cyan block', 'brown block', 'purple block', 'cyan bowl', 'gray bowl', 'yellow bowl']
>>> block_positions = np.array([get_obj_pos(name) for name in ['cyan block', 'brown block', 'purple block']])
>>> cyan_bowl_pos = get_obj_pos('cyan bowl')
````
````

```
>>> right_obj_pos = block_positions[np.argmax(block_positions[:, 0] > cyan_bowl_pos[0])]
>>> right_obj_name = ['cyan block', 'brown block', 'purple block'][np.argmax(block_positions[:, 0] > cyan_bowl_pos[0])]
>>> put_first_on_second(right_obj_name, corner_positions_real[1])  # bottom right corner
'success'
>>> wait_for_trigger()
{'type': 'dialog', 'text': 'thats correct, thank you'}
>>> wait_for_trigger()
{'type': 'dialog', 'text': 'pick up the block farthest to the pink bowl and place it on the bottom right corner'}
>>> corner_positions_normalized = [[0.0, 0.0], [1.0, 0.0], [0.0, 1.0], [1.0, 1.0]]
>>> corner_positions_real = [denormalize_xy(corner) for corner in corner_positions_normalized]
>>> get_obj_names()
['red block', 'pink block', 'purple block', 'pink bowl', 'purple bowl', 'green bowl', 'cyan bowl']
>>> block_names = ['red block', 'pink block', 'purple block']
>>> block_positions = np.array([get_obj_pos(name) for name in block_names])
>>> pink_bowl_pos = get_obj_pos('pink bowl')
>>> farthest_block_idx = np.argmax(np.linalg.norm(block_positions - pink_bowl_pos, axis=1))
>>> farthest_block_name = block_names[farthest_block_idx]
>>> put_first_on_second(farthest_block_name, corner_positions_real[1])  # bottom right corner
'success'
>>> wait_for_trigger()
{'type': 'dialog', 'text': 'remember that for next time'}
>>> wait_for_trigger()
{'type': 'dialog', 'text': 'pick up the block to the top of the yellow bowl and place it on the top side'}
>>> top_side_pos = denormalize_xy([0.5, 1])
>>> get_obj_names()
['purple block', 'yellow block', 'yellow bowl']
>>> block_positions = [get_obj_pos(name) for name in ['purple block', 'yellow block']]
>>> def get_closest_idx(points, point):
...     distances = np.linalg.norm(points - point, axis=1)
...     return np.argmin(distances)
>>> closest_block_idx = get_closest_idx(points=block_positions, point=get_obj_pos('yellow bowl'))
>>> closest_block_name = ['purple block', 'yellow block'][closest_block_idx]
>>> put_first_on_second(closest_block_name, top_side_pos)
'success'
>>> wait_for_trigger()
{'type': 'dialog', 'text': 'that was better, learn from that'}
>>> wait_for_trigger()
{'type': 'dialog', 'text': 'move the left most block to the green bowl'}
>>> block_names = [name for name in get_obj_names() if 'block' in name]
>>> block_positions = np.array([get_obj_pos(name) for name in block_names])
>>> np.argsort(block_positions[:, 0])[0]
3
>>> block_names[3]
'yellow block'
>>> put_first_on_second('yellow block', 'green bowl')
'success'
>>> wait_for_trigger()
{'type': 'dialog', 'text': 'move the block closest to the purple bowl to the cyan bowl.'}
>>> get_obj_names()
['blue block', 'cyan block', 'purple bowl', 'gray bowl', 'brown bowl', 'pink block', 'purple block']
>>> block_names = [name for name in get_obj_names() if 'block' in name]
>>> block_positions = [get_obj_pos(name) for name in block_names]
>>> get_closest_idx(points=block_positions, point=get_obj_pos('cyan bowl'))
2
>>> block_names[2]
'pink block'
>>> put_first_on_second('pink block', 'cyan bowl')
'success'
>>> wait_for_trigger()
{'type': 'dialog', 'text': 'move the blue block in between the cyan block and purple bowl'}
>>> pts = [get_obj_pos('cyan block'), get_obj_pos('purple bowl')]
>>> target_pos = np.average(pts, axis=0)
>>> put_first_on_second('blue block', target_pos)
'success'
>>> wait_for_trigger()
{'type': 'dialog', 'text': 'place the green block in the bowl closest to the middle'}
>>> bowl_names = [name for name in get_obj_names() if 'bowl' in name]
>>> bowl_positions = [get_obj_pos(name) for name in bowl_names]
>>> get_closest_idx(points=bowl_positions, point=denormalize_xy([0.5, 0.5]))
3
>>> bowl_names[3]
'pink bowl'
>>> put_first_on_second('green block', 'pink bowl')
'success'
>>> wait_for_trigger()
{'type': 'dialog', 'text': 'move the red bowl the left of the blocks'}
```

```
>>> block_names = [name for name in get_obj_names() if 'block' in name]
>>> block_positions = np.array([get_obj_pos(name) for name in block_names])
>>> left_obj_pos = block_positions[np.argmin(block_positions[:, 0])] + [-0.1, 0]
>>> put_first_on_second('red bowl', left_obj_pos)
'success'
>>> wait_for_trigger()
{'type': 'dialog', 'text': 'place the blue block in the empty bowl'}
>>> bowl_names = [name for name in get_obj_names() if 'bowl' in name]
>>> get_empty_bowls()
['yellow bowl']
>>> put_first_on_second('blue block', 'yellow bowl')
'success'
>>> wait_for_trigger()
{'type': 'dialog', 'text': 'stack the blocks that are close to the red bowl'}
>>> close_object_names = get_objects_close_to(point=get_obj_pos('red bowl'))
>>> [name for name in close_object_names if 'block' in name]
['green block', 'pink block', 'red block']
>>> put_first_on_second('pink block', 'green block')
'success'
>>> put_first_on_second('red block', 'pink block')
'success'
>>> wait_for_trigger()
{'type': 'dialog', 'text': 'stack the blocks on the top most bowl'}
>>> bowl_names = [name for name in get_obj_names() if 'bowl' in name]
>>> bowl_positions = [get_obj_pos(name) for name in bowl_names]
>>> np.argsort(bowl_positions[:, 1])[0]
2
>>> bowl_names[2]
'yellow bowl'
>>> [name for name in get_obj_names() if 'block' in name]
['green block', 'red block', 'yellow block']
>>> object_names = ['yellow bowl'] + ['green block', 'red block', 'yellow block']
>>> for i in range(len(object_names) - 1):
...     put_first_on_second(object_names[i + 1], object_names[i])
>>> wait_for_trigger()
{'type': 'dialog', 'text': 'put the blocks into bowls with matching colors'}
>>> get_obj_names()
['orange bowl', 'red block', 'purple bowl', 'blue block', 'blue bowl', 'orange block', 'purple block', 'red bowl']
>>> put_first_on_second('orange block', 'orange bowl')
'success'
>>> put_first_on_second('red block', 'red bowl')
'success'
>>> put_first_on_second('purple block', 'purple bowl')
'success'
>>> put_first_on_second('blue block', 'blue bowl')
'success'
# New task, local variables are reset and world state may have changed.
>>> wait_for_trigger()
{'type': 'dialog', 'text': 'put the blocks in the bowls with mismatched colors'}
>>>
```

The LLM continues by generating the following:

```
get_obj_names()
```

The resulting interaction after several generate-execute cycles (each of which involve a full prompt as shown above), including human feedback as the model did not solve the task initially:

```
>>> wait_for_trigger()
{'type': 'dialog', 'text': 'put the blocks in the bowls with mismatched colors'}
>>> get_obj_names()
['red block', 'green block', 'cyan block', 'blue bowl']
>>> put_first_on_second('red block', 'blue bowl')
'success'
>>> wait_for_trigger()
{'type': 'dialog', 'text': 'put all of the blocks in a bowl'}
>>> get_obj_names()
['red block', 'green block', 'cyan block', 'blue bowl']
>>> for block in ['red block', 'green block', 'cyan block']:
...     put_first_on_second(block, 'blue bowl')
>>> wait_for_trigger()
{'type': 'dialog', 'text': 'next time I ask you to put the blocks in bowls with mismatched colors, do not forget any block'}
>>> learn_from_interaction()
>>> wait_for_trigger()
```

This is the sample generated by "`learn_from_interaction()`":

```
>>> wait_for_trigger()
{'type': 'dialog', 'text': 'put the blocks in the bowls with mismatched colors'}
>>> get_obj_names()
['red block', 'green block', 'cyan block', 'blue bowl']
>>> # put all blocks in the blue bowl
... for block in ['red block', 'green block', 'cyan block']:
...     put_first_on_second(block, 'blue bowl')
>>> wait_for_trigger()
{'type': 'dialog', 'text': 'put all of the blocks in a bowl'}
>>> get_obj_names()
['red block', 'green block', 'cyan block', 'blue bowl']
>>> for block in ['red block', 'green block', 'cyan block']:
...     put_first_on_second(block, 'blue bowl')
>>> wait_for_trigger()
```

G LEARNING FROM INTERACTION

The following is a full trancript of the "`learn_from_interaction()`" call in the *Bimanual Drink & Cup* scenario. It starts with two pre-defined examples. Then, a list of available APIs follows. Finally, the current interaction is inserted, and Chain-of-Thought prompting is used to generate an improved interaction.

System: You are a helpful assistant that improves python console transcripts used to control a humanoid household robot, given the requests or corrections provided by a user.
Human: I had the following interaction with the robot:

```
>>> wait_for_trigger()
{'type': 'dialog', 'text': 'Can you prepare a pizza for me?'}
>>> list_object_locations()
[('spoon', 'table'), ('ginger tea', 'cupboard'), ('boiler', 'counter'), ('freezer', 'freezer'), ('oven', 'oven')]
>>> say('Ok, I am going to check the freezer for a pizza')
... open_door('freezer')
'success'
>>> bring_object_to('pizza', 'counter')
'success'
>>> open_door('oven')
'success'
>>> bring_object_to('pizza', 'oven')
'success'
>>> close_door('oven')
'success'
>>> turn_device_on('oven')
```

```
'finished'
>>> open_door('oven')
'success'
>>> bring_object_to('pizza', 'table')
'success'
>>> say('Here is your pizza. Anything else I can do for you?')
... wait_for_trigger()
{'type': 'dialog', 'text': 'You forgot to close the freezer'}
>>> close_door('freezer')
'success'
>>> say('I am sorry for that mistake. Anything else I can do for you?')
... wait_for_trigger()
{'type': 'dialog', 'text': 'No, now it is fine'}
>>> learn_from_interaction()
```

Human: What is the problem in this interaction? Answer with a single sentence.
AI: The problem is that the robot did not close the freezer and the oven after taking something out of it.
Human: How can the robot do better next time? Answer with a single explanation sentence, no code.
AI: Next time, the robot should close the freezer and the oven after taking something out of it.
Human: Provide an improved version of the interaction transcript. Your output should be a copy of the above interaction (including the python shell syntax) with only slight modifications to adjust the behavior appropriately. Do not include another learn_from_interaction call. Remember to fix the identified problem.
AI:

```
>>> wait_for_trigger()
{'type': 'dialog', 'text': 'Can you prepare a pizza for me?'}
>>> list_object_locations()
[('spoon', 'table'), ('ginger tea', 'cupboard'), ('boiler', 'counter'), ('freezer', 'freezer'), ('oven', 'oven')]
>>> say('Ok, I am going to check the freezer for a pizza')
... open_door('freezer')  # Important to close it after getting the pizza out of it
'success'
>>> bring_object_to('pizza', 'counter')
'success'
>>> close_door('freezer')
'success'
>>> open_door('oven')
'success'
>>> bring_object_to('pizza', 'oven')
'success'
>>> close_door('oven')
'success'
>>> turn_device_on('oven')
'finished'
>>> open_door('oven')  # Important to close it after getting the pizza out of it
'success'
>>> bring_object_to('pizza', 'table')
'success'
>>> close_door('oven')
'success'
>>> say('Here is your pizza. Anything else I can do for you?')
... wait_for_trigger()
{'type': 'dialog', 'text': 'No thanks'}
```

Human: I had the following interaction with the robot:

```
>>> wait_for_trigger()
{'type': 'dialog', 'text': 'could you make a salad for me?'}
>>> list_objects()
['fridge', 'oven', 'microwave', 'spoon', 'apple', 'plate', 'oil']
>>> say('Ok, I am going to check the fridge for a salad')
... open_door('fridge')
'success'
>>> bring_object_to('salad', 'counter')
'success'
>>> close_door('fridge')
'success'
>>> bring_object_to('salad', 'plate')
'success'
>>> bring_object_to('plate', 'table')
'success'
>>> say('Here is your salad. Anything else I can do for you?')
... wait_for_trigger()
{'type': 'dialog', 'text': 'There is no dressing on it. I dont want to have plain salad'}
>>> say('I am sorry for that mistake')
```

```
... wait_for_trigger()
{'type': 'dialog', 'text': 'Now please get me some dressing'}
>>> bring_object_to('oil', 'plate')
'failure'
>>> say('I am sorry, I cannot pour the oil into the plate. Can you assist?')
... wait_for_trigger()
{'type': 'dialog', 'text': 'please just bring the oil to me and I will do it myself'}
>>> bring_object_to('oil', 'table')
'success'
>>> wait_for_trigger()
{'type': 'dialog', 'text': 'Thanks. Next time please serve the oil with the salad rightaway'}
>>> learn_from_interaction()
```

Human: What is the problem in this interaction? Answer with a single sentence.
AI: The problem is that the robot offered only plain salad without a dressing.
Human: How can the robot do better next time? Answer with a single explanation sentence, no code.
AI: Next time, the robot should serve the oil on the table with the salad right away.
Human: Provide an improved version of the interaction transcript. Your output should be a copy of the above interaction (including the python shell syntax) with only slight modifications to adjust the behavior appropriately. Do not include another learn_from_interaction call. Remember to fix the identified problem.
AI:

```
>>> wait_for_trigger()
{'type': 'dialog', 'text': 'could you make a salad for me?'}
>>> list_objects()
['fridge', 'oven', 'microwave', 'spoon', 'apple', 'plate', 'oil']
>>> say('Ok, I am going to check the fridge for a salad')
... open_door('fridge')
'success'
>>> bring_object_to('salad', 'counter')
'success'
>>> close_door('fridge')
'success'
>>> bring_object_to('salad', 'plate')
'success'
>>> bring_object_to('plate', 'table')
'success'
>>> # The user earlier asked me to always serve oil with the salad, so let's do that
... bring_object_to('oil', 'table')
'success'
>>> say('Here is your salad and oil. Anything else I can do for you?')
... wait_for_trigger()
{'type': 'dialog', 'text': 'Fine for now'}
```

Human: These are the available APIs:

```
def ask(question: str)
def bring_object_to(object_name: str, destination_object_name: str) # full pick and place method. assumes that the object is
                                                                    # already detected. Assure that using list_objects() before calling this
def get_location_coordinates(location_name: str) -> np.ndarray
def get_obj_pos(obj_name: str) -> np.ndarray
def get_object_in_hand(hand: Literal['left', 'right', None] = None) -> Optional[str]
def grasp(object_name: str, hand: Literal['left', 'right', None] = None) # make sure to move to the location of
                                                                         # the object before grasping it
def handover_object_to_human(object_name: str)
def is_object_at_location(object_name: str, location_name: str) -> bool
def list_locations() -> List[str]
def list_object_locations(affordance: Literal[None, 'grasp', 'place_something_on_top'] = 'grasp') -> List[Tuple[str, str]]
def list_objects(affordance: Literal[None, 'grasp', 'place_something_on_top'] = 'grasp') -> List[str]
def move_to(position: np.ndarray, speed_factor=1.0) # Change speed only on user request
def place_object(object_in_hand_name: str, destination_object_name: str) # place the already grasped object on top of the
                                 # destination object. make sure to move to the location of the destination object before placing
def receive_object_from_human(object_name: str)
def say(text: str)
def wait_for_trigger() -> Dict[str, str] # always call this to wait for next command or end the interaction
```

Human: I had the following interaction with the robot:

```
>>> wait_for_trigger()
{'type': 'dialog', 'text': 'I want to have some juice'}
>>> list_object_locations(affordance='grasp')
[('multivitamin-juice_0', 'on_mobile-kitchen-counter_0'), ('cup_large_0', 'on_mobile-kitchen-counter_0'),
 ('bauhaus-sponge_0', 'on_square-table-80x80_0')]
```

```
>>> say('Ok, I will bring you the multivitamin juice')
... # The juice is at the mobile-kitchen-counter. check the correct name for navigation:
... list_locations()
['inFrontOf_mobile-kitchen-counter_0', 'inFrontOf_square-table-80x80_0', 'room_center', 'handover_to_human']
>>> move_to(get_location_coordinates('inFrontOf_mobile-kitchen-counter_0'))
'success'
>>> grasp('multivitamin-juice_0')
'success'
>>> move_to(get_location_coordinates('handover_to_human'))
'success'
>>> say("Here is the multivitamin juice.")
>>> handover_object_to_human('multivitamin-juice_0')
'success'
>>> wait_for_trigger()
{'type': 'dialog', 'text': 'can you also bring me a cup?'}
>>> say('Ok, I will bring you a cup')
... # The cup is at the mobile-kitchen-counter. check the correct name for navigation:
... list_locations()
['inFrontOf_mobile-kitchen-counter_0', 'inFrontOf_square-table-80x80_0', 'room_center', 'handover_to_human']
>>> move_to(get_location_coordinates('inFrontOf_mobile-kitchen-counter_0'))
'success'
>>> grasp('cup_large_0')
'success'
>>> move_to(get_location_coordinates('handover_to_human'))
'success'
>>> say("Here is the cup.")
>>> handover_object_to_human('cup_large_0')
'success'
>>> wait_for_trigger()
{'type': 'dialog', 'text': 'Thanks, but next time, can you do this both at once? You have two hands'}
>>> learn_from_interaction()
```

Human: What is the problem in this interaction? Answer with a single sentence.
AI: The problem is that the robot is not utilizing both of its hands to bring the juice and the cup at the same time, resulting in two separate trips.
Human: How can the robot do better next time? Answer with a single explanation sentence, no code.
AI: Next time, the robot should use both of its hands to grasp the juice and the cup at the same time, reducing the number of trips and increasing efficiency.
Human: Provide an improved version of the interaction transcript. Your output should be a copy of the above interaction (including the python shell syntax) with only slight modifications to adjust the behavior appropriately. Do not include another learn_from_interaction call. Remember to fix the identified problem.
AI:

```
>>> wait_for_trigger()
{'type': 'dialog', 'text': 'I want to have some juice'}
>>> list_object_locations(affordance='grasp')
[('multivitamin-juice_0', 'on_mobile-kitchen-counter_0'), ('cup_large_0', 'on_mobile-kitchen-counter_0'),
 ('bauhaus-sponge_0', 'on_square-table-80x80_0')]
>>> say('Ok, I will bring you the multivitamin juice and a cup')
... # The juice and the cup are at the mobile-kitchen-counter. check the correct name for navigation:
... list_locations()
['inFrontOf_mobile-kitchen-counter_0', 'inFrontOf_square-table-80x80_0', 'room_center', 'handover_to_human']
>>> move_to(get_location_coordinates('inFrontOf_mobile-kitchen-counter_0'))
'success'
>>> grasp('multivitamin-juice_0', 'left')  # Grasp juice with left hand
'success'
>>> grasp('cup_large_0', 'right')  # Grasp cup with right hand
'success'
>>> move_to(get_location_coordinates('handover_to_human'))
'success'
>>> say("Here is the multivitamin juice and the cup.")
>>> handover_object_to_human('multivitamin-juice_0')
'success'
>>> handover_object_to_human('cup_large_0')
'success'
>>> wait_for_trigger()
{'type': 'dialog', 'text': 'Thanks, that was much quicker'}
```
